# Bioinspired Tumor‐Targeting and Biomarker‐Activatable Cell‐Material Interfacing System Enhances Osteosarcoma Treatment via Biomineralization

**DOI:** 10.1002/advs.202302272

**Published:** 2023-05-21

**Authors:** Xiao Yang, Simin Gao, Boguang Yang, Zhinan Yang, Feng Lou, Pei Huang, Pengchao Zhao, Jiaxin Guo, Huapan Fang, Bingyang Chu, Miaomiao He, Ning Wang, Anthony Hei Long Chan, Raymond Hon Fu Chan, Zuankai Wang, Liming Bian, Kunyu Zhang

**Affiliations:** ^1^ Department of Biomedical Engineering The Chinese University of Hong Kong Shatin Hong Kong 999077 China; ^2^ Department of Mechanical Engineering The Hong Kong Polytechnic University Hong Kong 999077 China; ^3^ Hong Kong Centre for Cerebro‐Cardiovascular Health Engineering (COCHE) Shatin Hong Kong 999077 China; ^4^ Department of Mechanical Engineering City University of Hong Kong Kowloon Hong Kong 999077 China; ^5^ Department of Otorhinolaryngology and Sleep Medicine Center West China School of Public Health and West China Forth Hospital Sichuan University Chengdu 610065 China; ^6^ State Key Laboratory of Oral Diseases West China Hospital of Stomatology Sichuan University Chengdu 610041 China; ^7^ School of Chemistry and Chemical Engineering Frontiers Science Center for Transformative Molecules Shanghai Jiao Tong University Shanghai 200240 China; ^8^ Department of Orthopaedics and Traumatology Faculty of Medicine The Chinese University of Hong Kong Shatin Hong Kong 999077 China; ^9^ Institute of Functional Nano and Soft Materials (FUNSOM) Jiangsu Key Laboratory for Carbon Based Functional Materials and Devices Soochow University Suzhou Jiangsu 215123 China; ^10^ State Key Laboratory of Biotherapy and Cancer Center West China Hospital Sichuan University Collaborative Innovation Center for Biotherapy Chengdu Sichuan 610065 China; ^11^ Analytical and Testing Center Sichuan University Chengdu 610065 China; ^12^ College of Pharmacy and Bioengineering Chongqing University of Technology Chongqing 400054 China; ^13^ Centre for Nature‐Inspired Engineering City University of Hong Kong Kowloon Hong Kong 999077 China; ^14^ School of Biomedical Sciences and Engineering South China University of Technology Guangzhou International Campus Guangzhou 511442 China; ^15^ National Engineering Research Center for Tissue Restoration and Reconstruction South China University of Technology Guangzhou 510006 China

**Keywords:** bioinspired material, biomineralization, self‐assembly, supramolecular hydrogel, tumor inhibition

## Abstract

Osteosarcoma is an aggressive malignant tumor that primarily develops in children and adolescents. The conventional treatments for osteosarcoma often exert negative effects on normal cells, and chemotherapeutic drugs, such as platinum, can lead to multidrug resistance in tumor cells. Herein, this work reports a new bioinspired tumor‐targeting and enzyme‐activatable cell‐material interface system based on DDDEEK‐pY‐phenylboronic acid (SAP‐pY‐PBA) conjugates. Using this tandem‐activation system, this work selectively regulates the alkaline phosphatase (ALP) triggered anchoring and aggregation of SAP‐pY‐PBA conjugates on the cancer cell surface and the subsequent formation of the supramolecular hydrogel. This hydrogel layer can efficiently kill osteosarcoma cells by enriching calcium ions from tumor cells and forming a dense hydroxyapatite layer. Owing to the novel antitumor mechanism, this strategy neither hurts normal cells nor causes multidrug resistance in tumor cells, thereby showing an enhanced tumor treatment effect than the classical antitumor drug, doxorubicin (DOX). The outcome of this research demonstrates a new antitumor strategy based on a bioinspired enzyme‐responsive biointerface combining supramolecular hydrogels with biomineralization.

## Graphical abstract

A tumor‐targeting and enzyme‐activatable cell‐material interface system based on DDDEEK‐pY‐phenylboronic acid (SAP‐pY‐PBA) conjugates are shown to efficiently kill osteosarcoma cells by enriching calcium ions from tumor cells and forming a dense hydroxyapatite layer. To sum up, this system targeted to cancer cells surfaces to induce specific cell calcification as a promising strategy for developing alternative therapeutics to treat osteosarcoma.

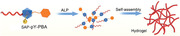

## 1 Introduction

Osteosarcoma, derived from osteoid tissues, is an aggressive malignant neoplasm that arises from transformed primitive cells of mesenchymal origin and triggers osteoblastic differentiation and malignant osteoid development, especially among children and adolescents.^[^ ^1^ ^]^ Currently, the conventional treatments for osteosarcoma are limited to chemotherapy, radiotherapy, and surgery.^[^ ^2^ ^]^ Unfortunately, these treatments exhibit major limitations, such as toxicity in normal cells and multidrug resistance in tumor cells.^[^ ^3^ ^]^ Polypeptides can self‐assemble into different nanostructures and usually exhibit good biocompatibility and bioactivity, hence peptide‐based nanomaterials are promising functional vehicles for drug delivery, immune manipulation, and regenerative medicine.^[^ ^4^ ^]^ Enzyme‐instructed self‐assembly capitalizes on enzymes overexpressed in cancer to generate self‐assembled peptide nanostructures in situ and demonstrates promising potential in cancer diagnosis and treatment.^[^ ^5^ ^]^ Furthermore, the triggered self‐assembly of peptides can be achieved on cell surfaces through specific interactions, enabling the development of selectively triggered supramolecular structure formation in the pericellular space of targeted cells.^[^ ^6^ ^]^

Targeted nanodrug delivery systems can increase the local drug concentration in a tumor, reduce the cytotoxicity in healthy tissues, and improve overall therapeutic efficacy.^[^ ^7^ ^]^ According to the reference,^[^ ^8^ ^]^ overexpression of sialic acid (SA) on cell membranes is associated with malignant and metastatic phenotypes in various cancers. Therefore, SA is an important molecular target for diagnostic and therapeutic approaches. Deshayes et al. developed PBA‐PEG‐PLGA micellar nanocarriers loaded with dichloro (1,2‐diaminocyclohexane) Platinum (II) to specifically target SA which is typically overexpressed on the surface of cancer cells.^[^ ^9^ ^]^ Wang et al. chose phosphoryl tyrosine (pY) as a building block for designing self‐assembling precursors. This molecule is an evolutionarily optimized domain known to be dephosphorylated by alkaline phosphatase (ALP), and thus pY‐cholesterol can selectively kill platinum‐resistant ovarian cancer cells that overexpress ALP.^[^ ^10^ ^]^ Moreover, Zhao et al. proposed a drug‐free cancer cell‐targeting calcification strategy based on folate molecules to concentrate Ca^2+^ and induce the selective calcification and death of cancer cells.^[^ ^11^ ^]^ This strategy demonstrates significantly lower side effects and does not cause multidrug resistance. Our previous work demonstrated that the DDDEEK peptide sequence bioinspired by salivary acquired pellicle (SAP) can strongly absorb mineral ions. We thereby designed a series of DDDEEK‐modified functional molecules including polymers, polyphenols, and polypeptides, and these functional molecules could adhere to different interfaces and induce the activation of biomineralization, antibacterial, antifouling, and cell adhesion.^[^ ^12^ ^]^

**Figure 1:**
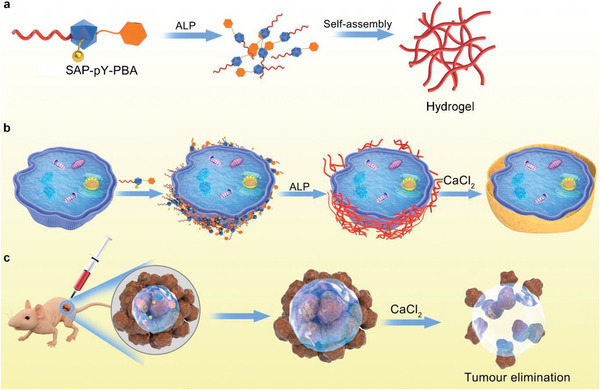
Schematic of a) SAP‐pY‐PBA can cut off phosphate group by alkaline phosphatase (ALP) and self‐assemble to the hydrogel. b) SAP‐pY‐PBA can anchor to the UMR106 cell surface and dephosphorylate by ALP secreted by UMR106 and form supramolecular hydrogel on the cell surface. After CaCl_2_ treatment, a calcium mineral layer form on the UMR106 cell surface. c) SAP‐pY‐PBA+CaCl_2_ can treat osteosarcoma in vivo.

Considering these aforementioned studies, we prepared a tumor‐targeting and biomarker‐activable l‐type DDDEEK‐pY‐phenylboronic acid (SAP‐pY‐PBA) conjugate. The PBA molecule recognizes SA molecules overexpressed on cancer cell surfaces, and the DDDEEK peptide induces biomineralization and then the death of cancer cells.^[^ ^13^ ^]^ To selectively target osteosarcoma cells, we inserted the enzyme‐activatable pY domain between DDDEEK and PBA (**Figure** **1**a). ALP overexpressed by osteosarcoma cells cleaves the phosphate group of pY, thereby initiating the hydrophobic and electrostatic interaction‐induced self‐assembly of the supramolecular conjugates into a hydrogel coating layer on cell surface (Figure 1b), which may further lead to the apoptosis of the cells. We further demonstrate that the combined administration of SAP‐pY‐PBA and CaCl_2_ improved the anticancer efficacy compared with the classical antitumor drug, doxorubicin (DOX), in an animal model (Figure 1c). We believe that the SAP‐pY‐PBA conjugate is a promising example of bioinspired tumor‐targeting and biomarker‐activatable cell‐material interactive systems that offers unique therapeutic advantages not found in conventional treatment.

## 2 Results and Discussion

### 2.1 ALP Triggers Self‐Assembly of the SAP‐pY‐PBA Supramolecular Hydrogels

**Figure 2:**
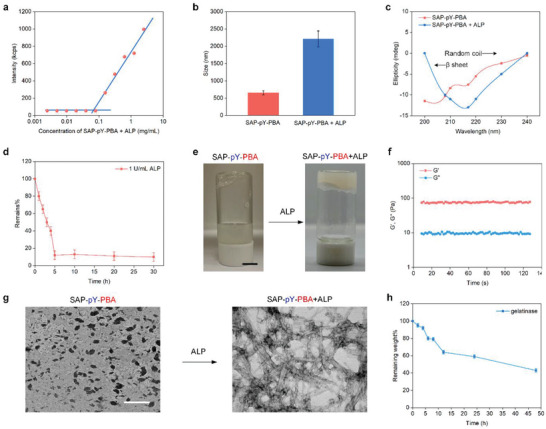
The SAP‐pY‐PBA conjugates form supramolecular hydrogels after alkaline phosphatase (ALP) triggered dephosphorylation. a) The critical micelle concentration (CMC) of SAP‐pY‐PBA+ALP (The concentration of ALP is 1 U mL^−1^). b) The particle sizes of SAP‐pY‐PBA and SAP‐pY‐PBA+ALP in deionized water were determined by dynamic light scattering (DLS). c) Circular dichroism (CD) spectrum of SAP‐pY‐PBA before and after adding ALP. d) Rapid dephosphorylation of SAP‐pY‐PBA by ALP. e) Photographs of the solution‐to‐hydrogel transition of SAP‐pY‐PBA+ALP hydrogel triggered by the addition of ALP. Scale bar = 0.5 cm. f) Time sweeps of dynamic rheology study of SAP‐pY‐PBA+ALP. g) Representative transmission electron microscopy (TEM) images of SAP‐pY‐PBA and SAP‐pY‐PBA+ALP hydrogel. Scale bar = 200 nm. h) The degradation rate of SAP‐pY‐PBA hydrogel, where the extent of degradation was determined as a percentage of the remaining weight. Data are expressed as the mean ± SD, *n* = 5.

After entering biological systems, peptides need to be degraded or cleared from the body after their action. Slower degradation of D‐amino acids leads to tissue accumulation, leading to side effects such as damage. Most importantly, D‐amino acids induce an immune response and rarely cross‐react with l‐enantiomers.^[^ ^14^ ^]^ Therefore, the amino acids used in this article are all l‐type. To fabricate the enzyme‐responsive SAP‐pY‐PBA conjugates, we chemically conjugated peptide DDDEEK to pY, a short peptide that can be dephosphorylated by ALP (Figure S1 and S2). Then, PBA, a small molecule that can target SA on the cell surface, was used to further modify DDDEEK‐pY to yield SAP‐pY‐PBA. First, we used mass spectrometry to verify that ALP can remove the phosphate group of SAP‐pY‐PBA. Before and after the addition of ALP, the relative molecular mass was 1111.4 and 1030.9, respectively, and the difference can only be attributed to the mass of a phosphate group (Figure S3 and S4). This result indicated that ALP can effectively remove the phosphate group of SAP‐pY‐PBA. As shown in **Figure** **2**a, the intensity of the SAP‐pY‐PBA+ALP group showed a dramatic increase when the concentration of SAP‐pY‐PBA is approximately 0.1 mg mL^−1^, revealing the critical micelle concentration (CMC) of this group. We also used dynamic light scattering (DLS), rheology, circular dichroism (CD), and Fourier‐transform infrared (FTIR) spectroscopy to further verify this finding. The size of the SAP‐pY‐PBA+ALP group was threefold that of the SAP‐pY‐PBA group (Figure 2b), and the CD data indicated that the secondary structure of SAP‐pY‐PBA+ALP was transformed from random coils to *β* sheets after the addition of ALP (Figure 2c). Compared with the SAP‐pY‐PBA group, the phosphate characteristic peaks of 960, 1040, and 1090 cm^−1^ were disappeared in the SAP‐pY‐PBA+ALP group (Figure S5), indicating the successful cleavage of the phosphate group. The dephosphorylation process lasted approximately 5 h (Figure 2d), and the hydrogel formation can be observed after that (Figure 2e). The G’ of the SAP‐pY‐PBA+ALP group was around 10^2^ Pa and larger than the G″ (Figure 2f), revealing the successful hydrogel formation of the SAP‐pY‐PBA+ALP group (Figure 2e). Transmission electron microscopy (TEM) results further confirmed that a tangled network structure of nanofibers was developed after the addition of ALP (Figure 2g). We also investigated the biodegradability of the SAP‐pY‐PBA+ALP hydrogels. After 48 h of gelatinase catalysis, the weight of the SAP‐pY‐PBA+ALP hydrogels was 50% of the original value (Figure 2h). Thermogravimetric analysis (TG) was also used to analyze the thermal stability of SAP‐pY‐PBA and SAP‐pY‐PBA+ALP groups. As shown in Figure S6, the two groups have similar thermal stability. In summary, ALP can trigger the self‐assembly of the SAP‐pY‐PBA supramolecular hydrogels.

### 2.2 SAP‐pY‐PBA Selectively Induces Calcification on Osteosarcoma Cell Surface

**Figure 3:**
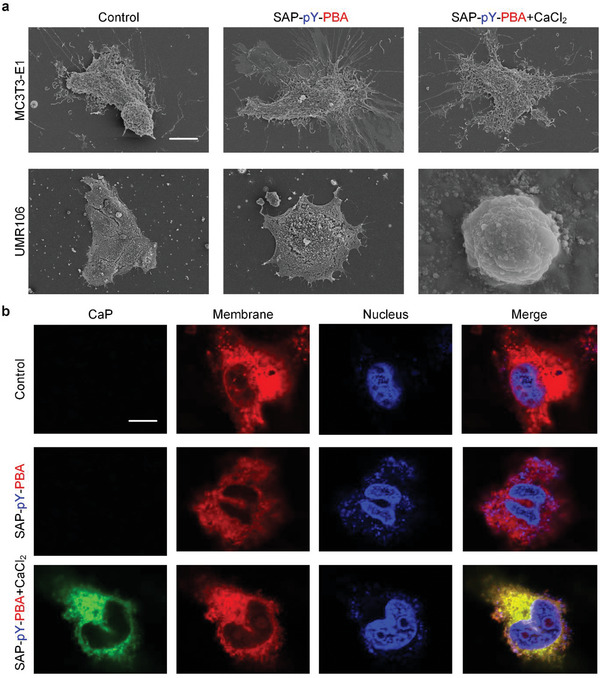
SAP‐pY‐PBA selectively induces calcification on the UMR106 cell surface. a) Scanning electron microscopy (SEM) imagine of MC3T3‐1E and UMR106 in the blank control, SAP‐pY‐PBA and SAP‐pY‐PBA+CaCl_2_ group. Scale bar: 20 µm. b) Confocal images of UMR106 stained with Calcein (CaP, green), Dil (cell membrane, red), and Hoechst (nuclei, blue) in the control, SAP‐pY‐PBA and SAP‐pY‐PBA+CaCl_2_ group. Scale bar = 20 µm.

To verify the effectiveness of PBA targeting SA in vitro, we treated rat osteosarcoma (UMR106) cells with 1 mg mL^−1^ free PBA to block the SA molecules on cancer cell surfaces prior to the addition of FITC‐labeled SAP‐pY‐PBA. As expected, no FITC fluorescence signal was detected in the PBA‐treated group (Figure S7). We further compared the aggregate of SAP‐pY‐PBA on different cell membranes by coculture of UMR106 or mouse osteoblast (MC3T3‐1E) cells with FITC‐labeled SAP‐pY‐PBA and a cell membrane dye (Dil). Although the green and red fluorescent signals matched well in both cells, the fluorescence intensity kept increasing from 4 to 24 h in UMR cells, while it remained consistently weak in MC3T3‐1E cells (Figure S8). These results indicate that SAP‐pY‐PBA can target tumor cells and accumulate on the surface of the cell membrane. In a cell calcification experiment, we cultured cells with SAP‐pY‐PBA for 24 h, followed by the CaCl_2_‐containing medium for another 24 h, in which the Ca^2+^ concentration refers to previous literature.^[^ ^11a^ ^]^ As shown in Figure S9, in the SAP‐pY‐PBA+CaCl_2_ group, the surface of UMR106 cells was covered with a layer of biominerals, while this phenomenon was not observed in the control or SAP‐pY‐PBA groups. For MC3T3‐1E cells, no obvious difference was observed among the three groups. We subsequently used scanning electron microscopy (SEM) to accurately observe the morphology of individual cells (**Figure** **3**a). In the control and SAP‐pY‐PBA groups, UMR106 cells exhibited a natural spreading morphology, but in the SAP‐pY‐PBA+CaCl_2_ group, many hierarchical structures consisting of biominerals grew on the cell surface. No significant difference among the three groups of MC3T3‐1E cells was observed. Energy‐dispersive X‐ray spectroscopy (EDS) was used to detect the surface composition of UMR106 and MC3T3‐1E cells in the SAP‐pY‐PBA+CaCl_2_ group, respectively. The results revealed the presence of 61.159 wt% Ca and 38.084 wt% P in the UMR106 cells, while only 17.941 wt% Ca and 14.198 wt% P was detected in the MC3T3‐1E cells (Figure S10). According to the EDS analysis, a calcium‐phosphorus ratio of 1.67 is hydroxyapatite.^[^ ^15^ ^]^ Subsequently, calcein (green), Dil (red), and Hoechst (blue) were used to stain CaP, cell membrane, and nuclei, respectively. The UMR106 cells in the SAP‐pY‐PBA+CaCl_2_ group emitted green fluorescence, while there was no green fluorescence detected in the MC3T3‐1E cells (Figure 3b and S11). These results indicate that although UMR106 and MC3T3‐1E cells secrete almost the same level of ALP,^[^ ^16^ ^]^ more SA (PBA receptor) on the surface of UMR106 cells contributes to the effective SAP‐pY‐PBA absorption,^[^ ^17^ ^]^ which may subsequently promote the formation of the calcified layer on the surface of UMR106 cells.

### 2.3 SAP‐pY‐PBA Exhibits Selective Antitumor Cytotoxicity

**Figure 4:**
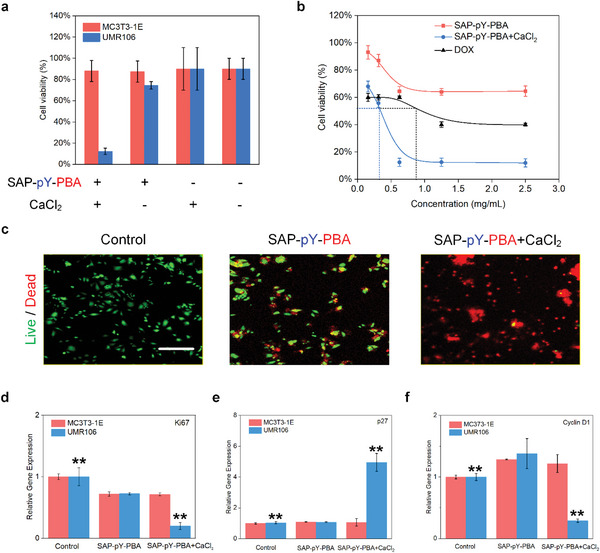
SAP‐pY‐PBA exhibits limited toxicity in MC3T3‐1E cells while can inhibit UMR106 cell activity by the arrest of cell cycle. a) Cell viability of MC3T3‐1E cell and UMR106 cell treated by SAP‐pY‐PBA (0.625 mg mL^−1^) in the presence of CaCl_2_ (1 mg mL^−1^) or not at 48 h. b) The half‐maximal inhibitory concentration (IC50) of SAP‐pY‐PBA, SAP‐pY‐PBA+CaCl_2_, and DOX against UMR106 cells at 48 h. c) Live/dead staining of UMR106 cell in control, SAP‐pY‐PBA and SAP‐pY‐PBA+CaCl_2_ groups, respectively. Scale bar = 500 µm. Quantification of d) Ki67, e) p27, f) Cyclin D1 by RT‐PCR in control, SAP‐pY‐PBA, and SAP‐pY‐PBA+CaCl_2_ groups, respectively. Data are expressed as the mean ± SD, *n* = 5. ^**^ *p* < 0.01.

Different concentrations (from 0.15625 to 2.5 mg mL^−1^) of SAP‐pY‐PBA were administered to examine its activity in MC3T3‐1E and UMR106 cell lines, respectively. As shown in Figure S12, neither SAP‐pY‐PBA nor SAP‐pY‐PBA+CaCl_2_ groups exhibited toxicity in MC3T3‐1E cells until the concentration was higher than 2.5 mg mL^−1^, while the DOX group started to show remarkable cytotoxicity at a much lower concentration (0.15625 µg mL^−1^). On the other hand, the viability of the UMR106 cells in the SAP‐pY‐PBA and SAP‐pY‐PBA+CaCl_2_ groups was approximately 80% and 20%, respectively, when the concentration of SAP‐pY‐PBA was 0.625 mg mL^−1^. As the viability of URM106 cells did not further decrease with the increased concentration of SAP‐pY‐PBA, this concentration was set as the working concentration hereafter (**Figure** **4**a). Notably, when treated with the 0.625 *µ*g mL^−1^ of DOX, the viability of UMR106 cells and MC3T3‐1E cells was about 35% and 40%, respectively, revealing the enhanced antitumor efficacy and reduced side effects of the SAP‐pY‐PBA+CaCl_2_ compared with DOX. We also investigated the antitumor effects of SAP‐pY‐PBA+CaCl_2_ on two human osteosarcoma cell lines, MG63 and U2OS, and a similar antitumor effect was observed (Figure S13). Moreover, the half‐maximal inhibitory concentration (IC_50_) of the SAP‐pY‐PBA+CaCl_2_ group was 0.32 mg mL^−1^ (48 h) (Figure 4b). We also performed live/dead staining to further verify these conclusions. As shown in Figure 4c and S15a, considerable green fluorescence (live cells) and a small amount of red fluorescence (dead cells) were observed in the UMR106 cells from control and SAP‐pY‐PBA groups, while only a small amount of live cells and considerable dead cells were observed in the SAP‐pY‐PBA+CaCl_2_ group. For MC3T3‐1E cells, a large amount of green fluorescence and a small amount of red fluorescence were detected in all three groups (Figure S14 and S15b). Consistently, the results of flow cytometry indicated the proportion of live cells reached 88.50% and 38.74% in the Q1 against MC3T3‐1E and UMR106 cells in the SAP‐pY‐PBA+CaCl_2_ group, respectively (Figure S16). Collectively, the SAP‐pY‐PBA+CaCl_2_ group exhibits limited toxicity in MC3T3‐1E cells while can inhibit UMR106 cell activity with greater potency than DOX. To further clarify the antitumor mechanism of SAP, we used the trimethylammonium‐diphenylhexatriene probe to detect the membrane fluidity of MC3T3‐1E and UMR106 cells after the calcification experiment. As shown in Figure S17a, the cell membrane fluidity of UMR106 cells was significantly decreased and the cell integrity of UMR106 cells was severely damaged, which was not observed in MC3T3‐1E cells. A hallmark of cancer cell metabolism is the ability to obtain essential nutrients from an often nutrient‐poor environment, which is closely related to tumor cells' reliance on aerobic glycolysis for energy (Warburg effect). Subsequently, to determine whether cancer cell calcification has an effect on energy supply and mitochondrial dysfunction in cancer cells, we performed glycolytic stress tests and oxygen consumption tests. The results revealed that SAP‐pY‐PBA+CaCl_2_‐induced cell calcification inhibited the glycolysis energy supply of UMR106 cells (Figure S17b). The oxygen consumption of mitochondria was also significantly reduced (Figure S17c).

To further understand the mechanism of UMR106 cell death induced by the SAP‐pY‐PBA+CaCl_2_ group, we evaluated the mRNA expression levels of cell proliferation and cell cycle progression‐related genes in tumor cells (Table S1). Ki67 expression is closely related to cell proliferation, and it cannot be detected in mitotic nonprogressing cells (G0).^[^ ^18^ ^]^ As shown in Figure 4d–f, the expression of Ki67 in UMR106 cells in the SAP‐pY‐PBA+CaCl_2_ group was significantly lower than those in the control group (*p* < 0.01). Meanwhile, the cell cycle phase is tightly regulated by cyclin‐cyclin‐dependent kinase protein complexes that can be blocked by CDK inhibitors, including p21/Cip1, p27/Kip1, and p57/Kip2.^[^ ^19^ ^]^ The relative gene expression of p27 in UMR106 cells in the SAP‐pY‐PBA+CaCl_2_ group was higher than that in the control and SAP‐pY‐PBA group (*p* < 0.01), while the cyclin D1 and cyclin E expression in UMR106 cells in the SAP‐pY‐PBA+CaCl_2_ group was lower than those in the control and SAP‐pY‐PBA group (Figure S18). Therefore, by regulating the expression of such molecules, the SAP‐pY‐PBA+CaCl_2_ group leads to the arrest of cell proliferation or cell cycle, which is of great significance to the prevention of tumorigenesis. Since the p53 and AKT pathways are closely related to cellular energy metabolism and glycolysis, we further studied the major molecular markers involved in AKT pathway after SAP‐pY‐PBA+CaCl_2_ treatment. Compared with the control and SAP‐pY‐PBA group, SAP‐pY‐PBA+CaCl_2_ upregulated the expression of PTEN and p53 and downregulated the expression of PI3K, p‐AKT, mTOR, PFKP, and PKM2 at protein levels (Figure S19). In conclusion, SAP‐pY‐PBA+CaCl_2_ may affect the malignant process of UMR106 cells by inhibiting the supply of energy and oxidative stress through AKT pathway.

### 2.4 SAP‐pY‐PBA Induces Selective Calcification and Inhibits Tumor Growth In Vivo

**Figure 5:**
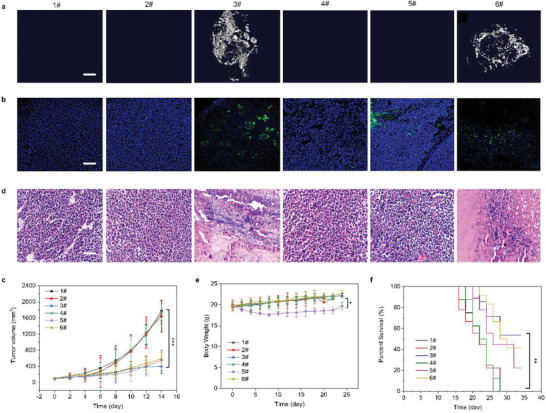
SAP‐pY‐PBA induces selective calcification and inhibits tumor growth in vivo. a) Micro‐CT images of calcification signals in tumors. b) Fluorescence images of a tumor slice stained by DAPI and TUNNEL. c) Average tumor‐growth kinetics of UMR106 tumor‐bearing mice in the control (1#), SAP‐pY‐PBA (2#), SAP‐pY‐PBA+CaCl2 (3#), CaCl2 (4#), DOX (5#), and SAP‐pY‐PBA via tail vein injection (6#) groups, respectively. The growth curves were plotted until the death of the first mouse. d) H&E staining of a tumor section. e) Body weights of mice, and f) survival curves corresponding to the mice in the control (1#), SAP‐pY‐PBA (2#), SAP‐pY‐PBA+CaCl2 (3#), CaCl_2_ (4#), DOX (5#), and SAP‐pY‐PBA via tail vein injection (6#) groups, respectively. Scale bar = 200 nm. Data are expressed as the mean ± SD, *n* = 5. ^***^ *p* < 0.001, ^**^ *p* < 0.01, ^*^ *p* < 0.05.

The FITC‐labeled peptide was injected via intravenous injection, and its tumor‐target ability was confirmed by in vivo imaging (mice) and ex vivo imaging (organs); organs included the tumor, heart, liver, spleen, lung, and kidney. As shown in Figure S20a, after the injection of SAP‐pY‐PBA, the fluorescence signals were detected all over the body initially, and then concentrated in the tumor area. To verify the effectiveness of PBA for SA targeting, we preprocessed SAP‐pY‐PBA with exogenous SA (SAP‐pY‐PBA+SA), and no FITC fluorescence signals were detected around tumor after 24 h. The same results were presented by the ex vivo images of the organs collected after 24 h (Figure S20b). In conclusion, when blocked by exogenous SA, SAP‐pY‐PBA lost its ability to target the tumor surface, indicating that SAP‐pY‐PBA does have targeting ability for SA on the tumor surface. To determine whether the SAP‐pY‐PBA+CaCl_2_ (3#) group can induce tumor calcification in vivo, UMR106 cells were injected into BALB/c or NOD/SCID mice to create the tumor model. When UMR106 cells were first injected into BALB/c mice, the tumors grew and then disappeared. Considering the role of immune function, the model was selected in immunodeficient mice (NOD/SCID mice). To induce calcification in vivo, an intratumoral injection of the SAP‐pY‐PBA solution followed by an intratumoral injection of the CaCl_2_ solution was performed. The intratumoral injection for osteosarcoma treatment has been reported by several other journal articles.^[^ ^20^ ^]^ Meanwhile, it has also been clinically used in the interventional therapy of osteosarcoma. And we also complemented the intravenous injection for preclinical in vivo treatment. After treatment, the color of tumors and surrounding tissues became darker (Figure S21), and significantly more calcification was detected in the SAP‐pY‐PBA+CaCl_2_ (3#) group compared with that in the control (1#), SAP‐pY‐PBA (2#), CaCl_2_ (4#), DOX (5#), and SAP‐pY‐PBA via tail vein injection (6#) groups (**Figure** **5**a). The representative optical images of isolated tumors from body after SAP‐pY‐PBA+CaCl_2_ treatment were shown in Figure S22. Based on these findings, we used TEM and energy dispersive X‐ray (EDX) to verify the calcification of tumor cells in vivo. As shown in Figure S23a, typical calcium and phosphorus signals (Ca:P = 1.67) were detected by EDX in the pericellular space, confirming the deposition of hydroxyapatite.^[^ ^15^ ^]^ However, no CaP was found in the tumor tissues in the control group; their intercellular spaces remained clear (Figure S23b). Taken together, we believe that the SAP kill the tumor through specific calcification both in vitro and in vivo. We also quantified the calcification by Micro‐CT software, and the calcium signaling in the SAP‐pY‐PBA+CaCl_2_ (3#) group was much greater than that in all the other groups (*p* < 0.01) (Figure S24a). Moreover, DAPI staining of the tumor slice showed the abnormal nuclear agglutination of the SAP‐pY‐PBA+CaCl_2_ (3#), DOX (5#) and SAP‐pY‐PBA via tail vein injection (6#) groups, while the nuclear morphology in the tumor tissue was normal in the control (1#), SAP‐pY‐PBA (2#), and CaCl_2_ (4#) groups (Figure 5b). TUNEL staining revealed many apoptotic cells (green fluorescence) in the SAP‐pY‐PBA+CaCl_2_ (3#), DOX (5#), and SAP‐pY‐PBA via tail vein injection (6#) groups, but no green fluorescence was detected in other groups (Figure S24b). The results indicate that the calcification of tumor tissue contributed to the death of cancer cells. It is worth mentioning that the intravenous injection also had the ability to inhibit tumor, which was slightly lower than that of intratumoral injection at the same administration concentration. Compared with the intratumoral injection, the tail vein injection may lose the effective drug dose due to enterohepatic circulation (or blood circulation), so the therapeutic effect of intratumoral injection is slightly better than that of tail vein. Therefore, an increased dose of the drug may be necessary for the intravenous therapy. Therefore, when we use intravenous therapy, we should consider increasing the dose of the drug that acts on the tumor.

Encouraged by the successful targeted calcification of tumor tissue by SAP‐pY‐PBA+CaCl_2_ (3#), we further evaluated its antitumor efficacy in UMR106 tumor mice. As shown in Figure 5c and Figure S25, the positive control using DOX (5#) administration exhibited a significant inhibitory effect on tumor growth compared with the other controls (tumor growth inhibition value (TGI), 68.41%). SAP‐pY‐PBA+CaCl_2_ (3#) exhibited an even higher antitumor effect, with a TGI value of 87.25%. H&E staining showed that both the SAP‐pY‐PBA+CaCl_2_ (3#) and DOX (5#) treatment reduced the number of cancer cells in the tumor tissues (Figure 5d), indicating that SAP‐pY‐PBA+CaCl_2_ (3#) successfully inhibited tumor growth in vivo. We also used neural network and multidimensional scaling (MDS) method to quantify H&E staining, and the convolutional neural network (CNN) numerically proved the significant difference among SAP‐pY‐PBA+CaCl_2_ and other groups (Figure S26).^[^ ^21^ ^]^ As expected, the mouse weight loss data indicated obvious side effects of DOX‐based chemotherapy, while SAP‐pY‐PBA+CaCl_2_ (3#) resulted in no significant mice weight loss (Figure 5e). Moreover, during the entire treatment, the survival rate of the mice in the SAP‐pY‐PBA+CaCl_2_ (3#) group was significantly higher than that of the mice in the DOX (5#) group, demonstrating another key advantage of SAP‐pY‐PBA+CaCl_2_ over conventional chemotherapy (Figure 5f). The control (1#), SAP‐pY‐PBA (2#), and CaCl_2_ (4#) had poorer survival than other groups due to ineffective control of tumor proliferation.

### 2.5 SAP‐pY‐PBA is Biocompatible In Vivo

We next used hematological and blood biochemical parameters and H&E staining to evaluate the biocompatibility of the SAP‐pY‐PBA+CaCl_2_ (3#). For hematology analysis, the platelet (PLT), white blood cell (WBC) count, red blood cell (RBC) count, hematocrit (HCT), mean corpuscular volume (MCV), and hemoglobin (HBC) in the SAP‐pY‐PBA+CaCl_2_ (3#) group were similar as that of the control (1#), SAP‐pY‐PBA (2#), CaCl_2_ (4#), and SAP‐pY‐PBA via tail vein injection (6#) groups (Figure S27a and S28).^[^ ^22^ ^]^ The amount of ALP, alanine aminotransferase (ALT), aspartate aminotransferase (AST), and blood urea nitrogen (BUN) in the SAP‐pY‐PBA+CaCl_2_ group were also similar to that of all the other groups (Figures S27b,c and S29). Moreover, the H&E staining of the SAP‐pY‐PBA+CaCl_2_ (3#) in major organs indicated no pathological changes compared with the control (1#), SAP‐pY‐PBA (2#), CaCl_2_ (4#), and SAP‐pY‐PBA via tail vein injection (6#) groups (Figures S27d and S30). These results imply the excellent biocompatibility of the SAP‐pY‐PBA+CaCl_2_, and minimal toxicity to nontumor tissues or normal organs in vivo.

In the context of developing antitumor hydrogels, our supramolecular hydrogel contains only one exogenous chemical compound, SAP‐pY‐PBA, and does not include any additional adjuvants or drugs. This one‐compound approach potentially eliminates concerns about possible short‐ or long‐term side effects commonly caused by synthetic and natural adjuvants or drugs. Systemic cancer treatment can induce side effects. This topical SAP‐pY‐PBA treatment reduced systemic toxicity, and no obvious side effects were observed in treated mice. These advantages highlight the important advantages of the supramolecular hydrogel drug delivery system.

## 3 Conclusion

In summary, we developed a bioinspired and biomarker‐activatable SAP‐pY‐PBA conjugate which can selectively self‐assemble and induce biomineralization on tumor cells, leading to tumor cell death. This strategy neither harms normal cells nor causes multidrug resistance in tumor cells. The treatment with SAP‐pY‐PBA+CaCl_2_ showed superior outcome compared with that of the treatment using a widely used antitumor drug DOX. These findings demonstrate a new antitumor strategy based on biomarker‐triggered supramolecular assembly and biomineralization.

## Conflict of Interest

The authors declare no conflict of interest.

## Supporting information

Supporting Information

## Data Availability

The data that support the findings of this study are available from the corresponding author upon reasonable request.
