# Supplementary material for: Bioinspired Tumor‐Targeting and Biomarker‐Activatable Cell‐Material Interfacing System Enhances Osteosarcoma Treatment via Biomineralization
Source: Adv Sci (Weinh). 2023 May 21;10(22):2302272. doi: 10.1002/advs.202302272 (PMC10401161; doi:10.1002/advs.202302272)
Supplement: Supplementary file 1 — Supporting Information [file ADVS-10-2302272-s001.pdf]

ADVANCED SCIENCE
Open Access

Supporting Information

for *Adv. Sci.*, DOI 10.1002/advs.202302272

Bioinspired Tumor-Targeting and Biomarker-Activatable Cell-Material Interfacing System Enhances Osteosarcoma Treatment via Biomineralization

Xiao Yang, Simin Gao, Boguang Yang, Zhinan Yang, Feng Lou, Pei Huang, Pengchao Zhao, Jiaxin Guo, Huapan Fang, Bingyang Chu, Miaomiao He, Ning Wang, Anthony Hei Long Chan, Raymond Hon Fu Chan, Zuankai Wang, Liming Bian* and Kunyu Zhang**

Supporting Information

A bioinspired tumor-targeting and biomarker-activatable cell-material interfacing system enhances osteosarcoma treatment via biomineralization

Xiao Yang, Simin Gao, Boguang Yang, Zhinan Yang, Feng Lou, Pei Huang, Pengchao Zhao, Jiaxin Guo, Huapan Fang, Bingyang Chu, Miaomiao He, Ning Wang, Anthony Hei Long Chan, Raymond Hon Fu Chan, Zuankai Wang*, Liming Bian*, Kunyu Zhang*

Experimental Section

Materials

All peptides are designed by us and synthesized by the Shanghai Botai Biotechnology company. All the other chemicals were purchased from Sigma-Aldrich and used without any purification.

Cell lines

The UMR106 cell lines (ATCC: CRL-1661) were donated by J. G. at the Chinese University of Hong Kong Department of Orthopaedics & Traumatology, M. H. at Sichuan University Analytical & Testing Center. UMR106 cells were grown in DMEM (Gibco, Invitrogen) supplemented with 10% FBS (Gibco, Invitrogen) and1% penicillin/streptomycin, PS at 37 °C in a 5% CO_2 incubator. The purchased MC3T3-E1 cells (Geneseed, Guangzhou, China) were cultured in α-MEM medium (containing 10% FBS and 1% penicillin/streptomycin, PS) at 37 °C in a 5% CO_2 incubator.

Animals

Female NOD/SCID mice (aged 4–6 weeks; Laboratory Animal Center, Sichuan University) were kept under a 12 h–12 h light-dark cycle at the Animal Care Facility with food and water ad libitum. Animal experiments were approved by the Animal Experimentation Ethics

Committee of Sichuan University (20200714037) and were carried out following the guidelines of the Animals Ordinance, China.

Synthesis of SAP-pY-PBA

The SAP-pY-PBA peptide conjugate was synthesized by solid-phase methods using standard Fmoc-chemistry. The peptide was confirmed using mass spectrometry and HPLC.[30]

Solution-to-gelation transition behavior

Solution-to-gelation transition experiments were performed by adding 1 U/mL ALP to 1 mg/mL SAP-pY-PBA solutions at 37℃ for 24 h. The solution-to-gelation transition behavior was tested using the inverted tube method. Rheological measurements of the materials were performed using a rotational rheometer (Malvern KINEXUS Lab+) at room temperature. For oscillatory time sweep experiments, the storage (G') and loss (G'') moduli were measured under constant strain (1%) and frequency (1 Hz).

MTT assay

The MTT method was used to detect cytotoxicity. MC3T3-1E and UMR106 cell lines were seeded on 96-well plates of 1×10^5 cells/well for 24 hours, then the medium was removed, and then the DMEM including different concentrations of SAP-pY-PBA peptide was added. After 24 hours, DMEM was removed, and cells were treated with DMEM containing 1 mg/mL $CaCl_2$ for 24 hours at 37°C in a humidified incubator with 5% CO_2 air. 20 μL of MTT (5 g/mL) was added and incubated at 37°C for 4 h. The supernatant per well was then replaced with 150 μL of dimethyl sulfoxide (DMSO). Absorbance was measured at OD = 590 nm. The cytotoxicity test was performed three times, and the average of the three measurements was taken. To ensure the reproducibility of the IC50 determination, we conducted six experiments and averaged all the experiments.

Targets calcification on the cell membrane

MC3T3-1E and UMR106 cell lines were seeded on 24-well plates and cultured with the DMEM containing SAP-pY-PBA peptide (0.625 mg/mL). After 24h, the medium was changed to the DMEM including 1 mg/mL $CaCl_2$ for 24 h. Then the calcified layer can be observed. The individual DMEM, DMEM (including extra 0.625 mg/mL SAP-pY-PBA peptide) and DMEM (including extra 1 mg/mL $CaCl_2$) alone treatment were used as comparison. After that, the

solution was removed carefully, the cells were incubated in the DMEM with 10% FBS at 37 °C with a 5% CO_2 air humidified incubator, or the cells were collected and then washed with PBS.

Optical microscopy and scanning electron microscope (SEM)

Optical microscopy was performed by an Eclipse NI-U microscope (Nikon, Japan). After calcification, the cells were fixed with 4% paraformaldehyde for 30 min at room temperature and dehydrated with gradient alcohol. SEM was performed by a SU8010 (Hitachi) equipped with a Ca-element mapping by EDS. The EDS was performed at an accelerating voltage of 15 kV.

Confocal laser scanning microscopy (CLSM)

The cells were stained with 5 μM of Dil red fluorescent probe for general cell membrane labeling (Ex. 549 nm, Em. 565 nm, Beyotime, China) for 5 min and 5 μg/mL Hoechst 33342 (Beyotime, China) for another 5 minutes. Then, the cells were rinsed with PBS for three times and subjected to CLSM (Nikon, Japan). All images were captured and analyzed with Image J (NIH). The cells were stained with Dil and Hoechst 33342 for general cell membrane and nucleus labeling, respectively, before the calcification treatment. After removing the staining solution, the cells were washed with PBS. Then the cells were seeded on 24-well plates and cultured with the DMEM containing SAP-pY-PBA peptide (0.625 mg/mL). After 24h, the medium was changed to the DMEM including 1 mg/mL $CaCl_2$ for 24 h. After then, the cells were fixed with 4% paraformaldehyde for 30 min at room temperature, and 1 μg/mL of Calcein was added, and the cells were rinsed with PBS and subjected to CLSM.

Gene expression analysis

For gene expression analysis, cells were collected and homogenized in TRIzol reagent, and RNA was extracted according to the manufacturer's instructions. The RNA concentration was measured by an ND-1000 spectrophotometer. Extract 100 ng of RNA from each sample and reverse transcribe into cDNA using RevertAid First Strand cDNA Synthesis Kit (Thermo). Real-time PCR was performed on an Applied Biosystems 7300 Real-Time PCR system using Taqman primers and probes (Table S1). The relative gene expression was calculated using the ΔΔCT method, where fold differences were calculated using the expression of $2^{\Delta\Delta CT}$.

Targeting of PBA *in vitro*

First, UMR106 cells were divided into 1 mg/mL PBA treatment group and no 1 mg/mL PBA treatment group for 30 mins. Then FITC labeled SAP-pY-PBA and the above cells were co-cultured for 2 hours. The fluorescence intensity was analyzed by confocal microscopy and Image J software.

Membrane fluidity

Specific cell membrane probe 1-(4-(trimethylamino) phenyl) -6-phenylhex-1,3, 5-triene (TMA-DPH) was used to detect the changes of cell membrane fluidity of MC3T3-1E and UMR106 after SAP-pY-PBA+$CaCl_2$ treatment. Briefly, cells were seeded into 96-well plates at a density of 1000 cells per well. After 48 h of SAP-pY-PBA+$CaCl_2$ treatment, PBS was washed three times, and 30 μl of TMADHP solution containing 3 μM TMADHP probe was added to each well and incubated at 37°C for 10 min. Excitation and emission wavelengths were 355 nm and 430 nm, respectively, and their intensities were measured on a microplate reader.

In vivo tumor models

To achieve calcification *in vivo*, 0.1 mL of cell suspension containing 5×10^6 UMR106 cells were injected into the right subaxillary area of each NOD/SCID mice to prepare the transplantable tumor. The mice were randomly divided into 6 groups (n = 6) when the tumors grew to a volume of approximately 60–100 mm^3.

In vivo tumor treatment by tumor tissue calcification

As listed in Table S2, the *in vivo* treatment included a 2-dosage intratumoral, intraperitoneal or tail vein injection, and the interval between the dosages was 20 minutes. The treatment was performed every other day. For the tumor tissue calcification assay, the mice were sacrificed after 7 treatments, and the tumors were detected by micro-CT (Quantum GX micro-CT), and the microscopic observation of CaP tumor was performed by CLSM observation in tumor slices. For the evaluation of anti-tumor efficacy, the tumor sizes were measured by a caliper every two days as well as the mice body weight. The tumor volume was calculated as the volume = (tumor length) × (tumor width)^2/2. To calculate the value of tumor growth inhibition (TGI), the tumor volumes on day 20 were recorded and calculated by the following equation,

Tumor growth inhibition (TGI) = $1-(V_{20}/V_0)_{experimental} / (V_{20}/V_0)_{control}$

where V_0 is the tumor volume on day 0 and V_{20}, the tumor volume on day 20.

Drug metabolism imaging

To evaluate the retention time and metabolism of the SAP-pY-PBA *in vivo*, FITC-NHS labeled SAP-pY-PBA were injected into the tail vein of mice subjected to the subcutaneous tumor model. Then, isoflurane was used to anaesthetize mice, and a small animal and a Xenogen IVIS Lumina imaging system (Perkin Elmer, Akron, OH, USA) were utilized to detect sites enriched with fluorescence over a continuous period. To investigate the metabolic pathway of SAP-pY-PBA and their distribution in the main organs of mice, the mice were euthanized within a fixed period after SAP-pY-PBA injection via the tail vein, and the main organs were collected to detect residual fluorescence signals with an IVIS system (Perkin Elmer, Akron, OH, USA).

Blood analysis and histology examinations

About 0.5 mL of the blood of each mouse was acquired for blood chemistry tests and complete blood panel analysis by eyeball extirpation at 14 days after treatment. And their major organs (heart, liver, spleen, lung, and kidneys) were harvested and fixed in 10% formalin solution. For hematology evaluation, we chose platelet count (PLT), white blood cells (WBC), red blood cells (RBC), hematocrit (HCT), mean corpuscular volume (MCV), hemoglobin (HGB), and as the markers. For serum biochemical analysis, the blood sample was centrifuged at 3,000 rpm for 15 minutes after standing at room temperature for 3 h. Then, the serum was collected from the supernatant and examined to evaluate serum levels of alanine aminotransferase (ALTL), aspartate aminotransferase (ASTL) and alkaline phosphatase (ALP). For histopathological tests, the tissue samples were embedded in paraffin blocks, sectioned into slices, and mounted onto the glass slides. After the hematoxylin-eosin (H&E) staining, the sections were examined by Eclipse NI-U microscope (Nikon, Japan).

Neural-network-based and multi-dimensional scaling method

Let X1, X2, X3, X4 be the image of the H&E staining of the tumor section in the control, SAP-pY-PBA, $CaCl_2$ and DOX groups respectively. Also, Let Y be that in the SAP-pY-PBA+$CaCl_2$ group. To analyze the difference among these groups more objectively and with high efficiency, the following neural-network-based method is used. Firstly, each image is cropped into sub-images $\boldsymbol{x}_i^j$. Each sub-image $\boldsymbol{x}_i^j$ has dimension 100x100. This process gives 1,440 sub-images for each image. Using the altogether 7,200 sub-images, a convolutional neural network (CNN) is built to classify between the SAP-pY-PBA+$CaCl_2$ group and all the other groups (taken as one class here). The CNN built has 2 hidden layers, each layer consists of a convolutional layer with 1-ring zero padding, 4 channels of 3x3 kernels running through the image with stride 1 and followed a max-pooling layer to decrease the size of the input data by half. Finally, a linear

fully connected layer is applied to give the classification result on the input data. The network achieves 92% accuracy on classifying between the SAP-pY-PBA+$CaCl_2$ and the "non-SAP-pY-PBA+$CaCl_2$" class on testing data, in which the database of 7,200 sub-images are randomly divided into training data and testing data in a 9:1 ratio. While the high accuracy of the CNN is promising, it is more important to obtain a numerical illustration of the difference between the SAP-pY-PBA+$CaCl_2$ and the "non-SAP-pY-PBA+$CaCl_2$" class. Note that the hidden layer of a CNN is usually referred to as the "features" of the input data. The features are hence used to generate the promised illustration. Let the CNN built be f, then f is a mapping that takes a 100x100x3-dimensional input and gives a 2-dimensional output, which is the probability that belongs to the SAP-pY-PBA+$CaCl_2$ class and the "non-SAP-pY-PBA+$CaCl_2$" class respectively. Here, the CNN $f=g \circ h$ can be divided into two mappings h, g, where h takes the input and outputs a 2,500-dimensional feature vector, and g takes this high-dimensional feature vector as input and outputs the 2-dimensional output as f. In this language, h ($\boldsymbol{x_i^j}$) gives the 2,500-dimensional feature vector for each sub-image $\boldsymbol{x_i^j}$. To make a meaningful visualization of the features, the traditional multi-dimensional scaling (MDS) technique is used. The MDS technique is useful for us to understand high-dimensional data visually. In our case, the MDS has been used to down-sample the 2,500-dimensional feature vector into a 2-dimensional vector, i.e., m (h ($\boldsymbol{x_i^j}$)) is a 2-dimensional vector.[1]

Statistical analysis

All data are shown as means ± SD via at least triplicate samples. Independent Student's t test and one-way analysis of variance (ANOVA) followed by a Tukey post hoc analysis were used to determine statistical significance between two or multiple groups, respectively. Statistical analyses were performed using SPSS (Statistical Package for the Social Sciences) 25.0, and a two-sided $p < 0.05$ was considered statistically significant.

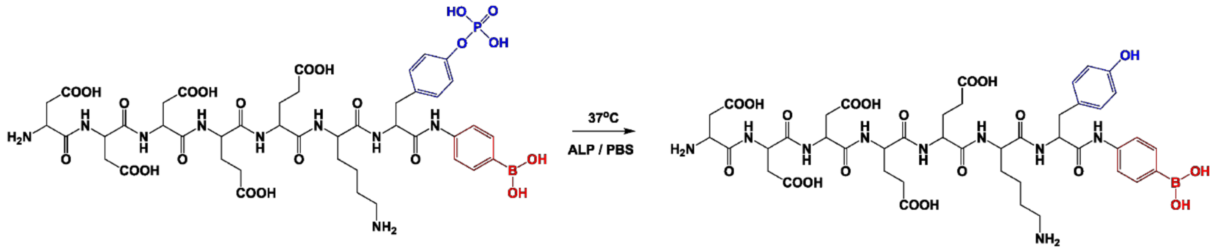

Figure S1. ALP-triggered dephosphorylation of SAP-pY-PBA.

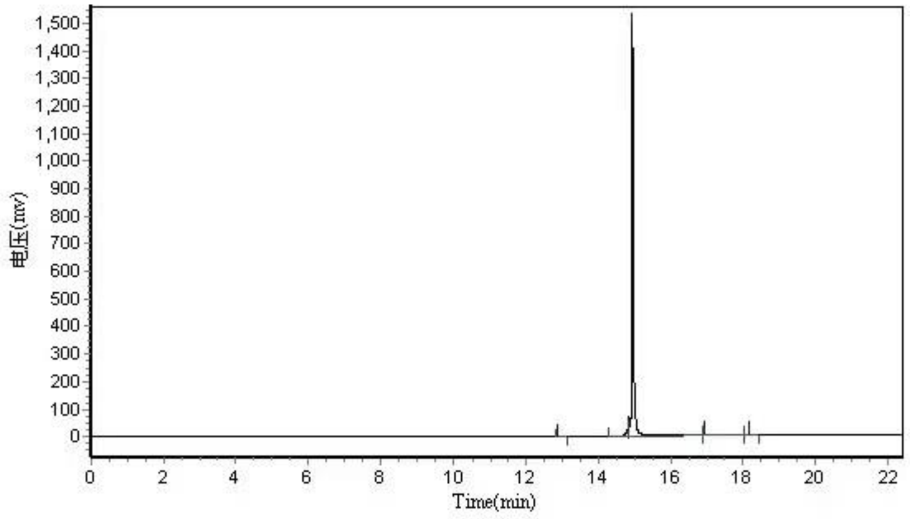

Results

Peak No.	Peak ID	Ret Time	Height	Area	Conc.
1		12.838	7.640	40.051	0.0009
2		14.802	19367.117	57746.055	1.3529
3		14.932	1481876.500	4179068.250	97.9082
4		16.915	681.771	29118.277	0.6822
5		18.163	140.638	2379.463	0.0557
**Total**			1502073.667	4268352.096	100.0000

Figure S2. HPLC of SAP-pY-PBA.

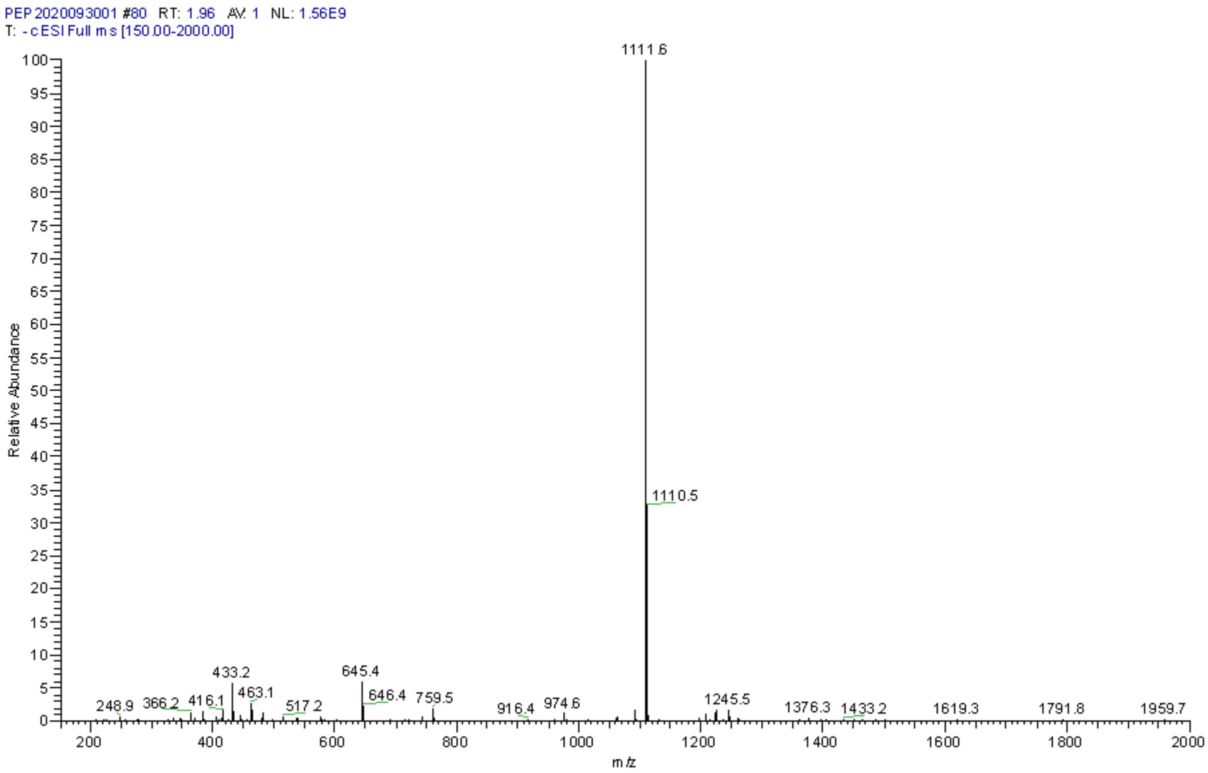

Figure S3. Mass spectra of SAP-pY-PBA.

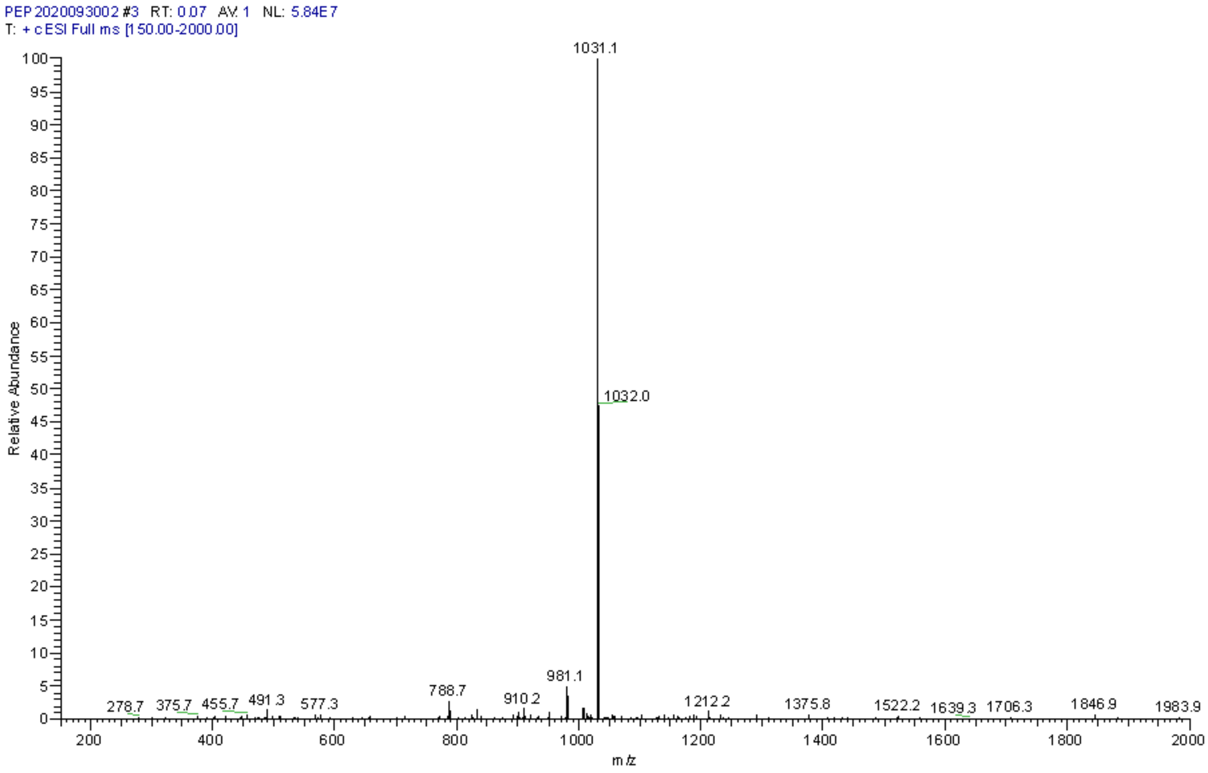

Figure S4. Mass spectra of SAP-pY-PBA+ALP.

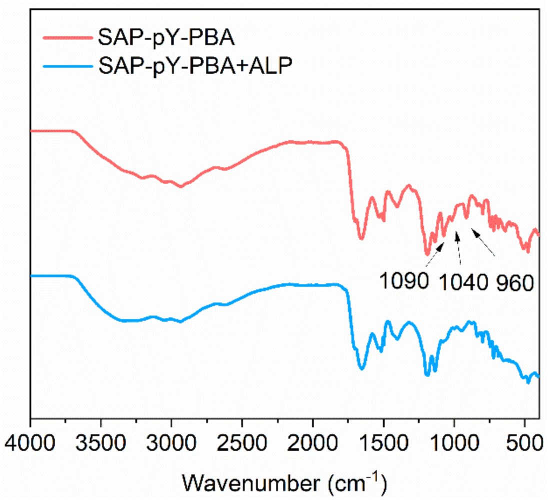

Figure S5. FTIR spectrum of SAP-pY-PBA before and after adding ALP.

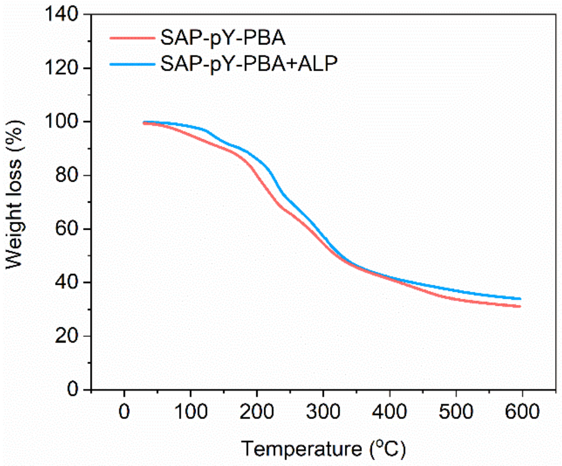

Figure S6. TG spectrum of SAP-pY-PBA before and after adding ALP.

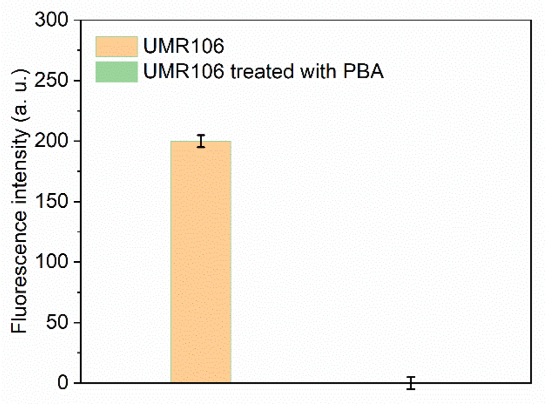

Figure S7. Fluorescence intensity of the UMR106 and UMR106 treated with PBA. Data are expressed as the mean ± SD, n = 5.

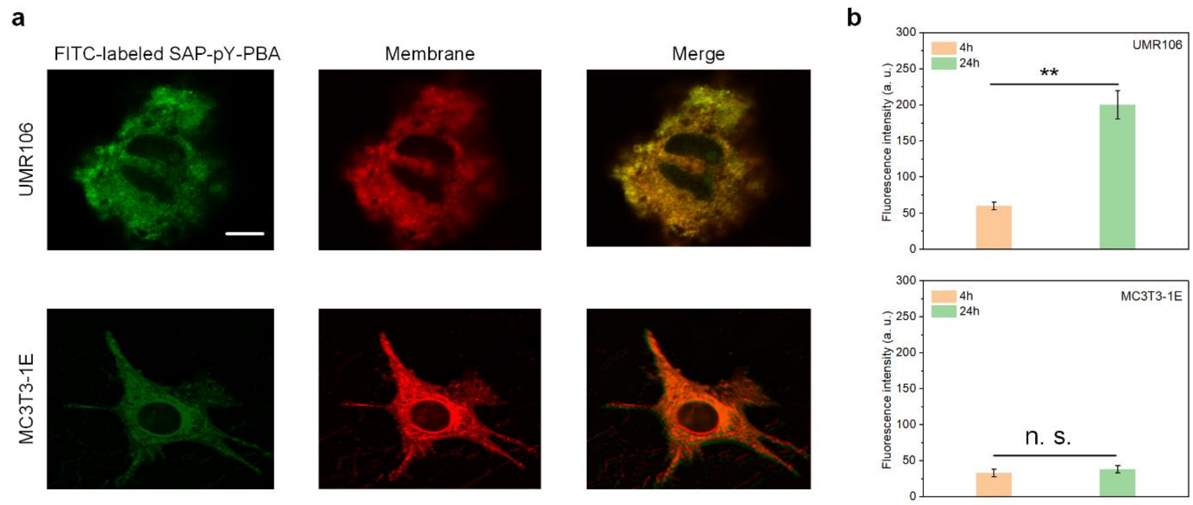

Figure S8. (a) Confocal images of UMR106 stained with FITC-labeled SAP-pY-PBA (FITC, green), Dil (cell membrane, red) in the SAP-pY-PBA group. Scale bar = 20 μm. (b) Fluorescence intensity of UMR106 treated with FITC-labeled SAP-pY-PBA at 4 and 24 hours. Data are expressed as the mean ± SD, n = 5.***p*<0.01.

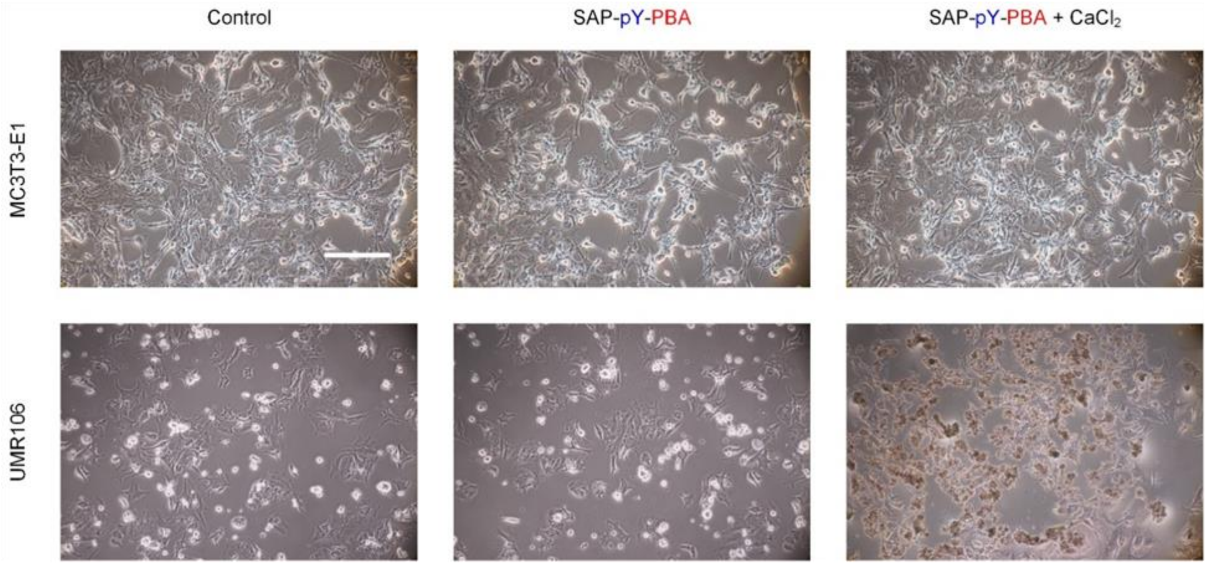

Figure S9. Optical images of MC3T3-1E and UMR106 cells in control, SAP-pY-PBA and SAP-pY-PBA+$CaCl_2$ groups, respectively. Scale bar: 0.1 μm.

Sample	Ca%	P%	Si%
MC3T3-E1	17.941 wt%	14.198 wt%	67.861 wt%
UMR106	61.159 wt%	38.084 wt%	0.757 wt%

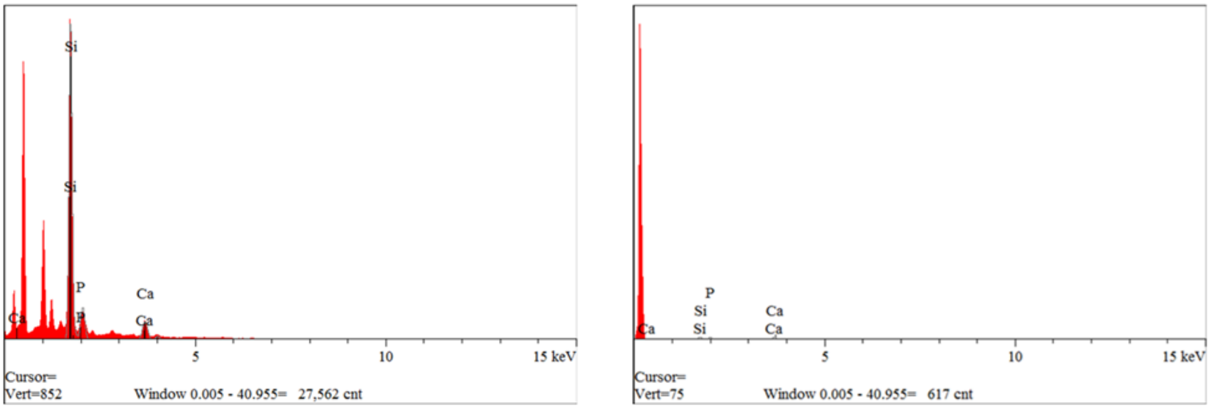

Figure S10. The Ca, P and Si content in the MC3T3-1E and UMR106 cells in the SAP-pY-PBA+$CaCl_2$ group, respectively.

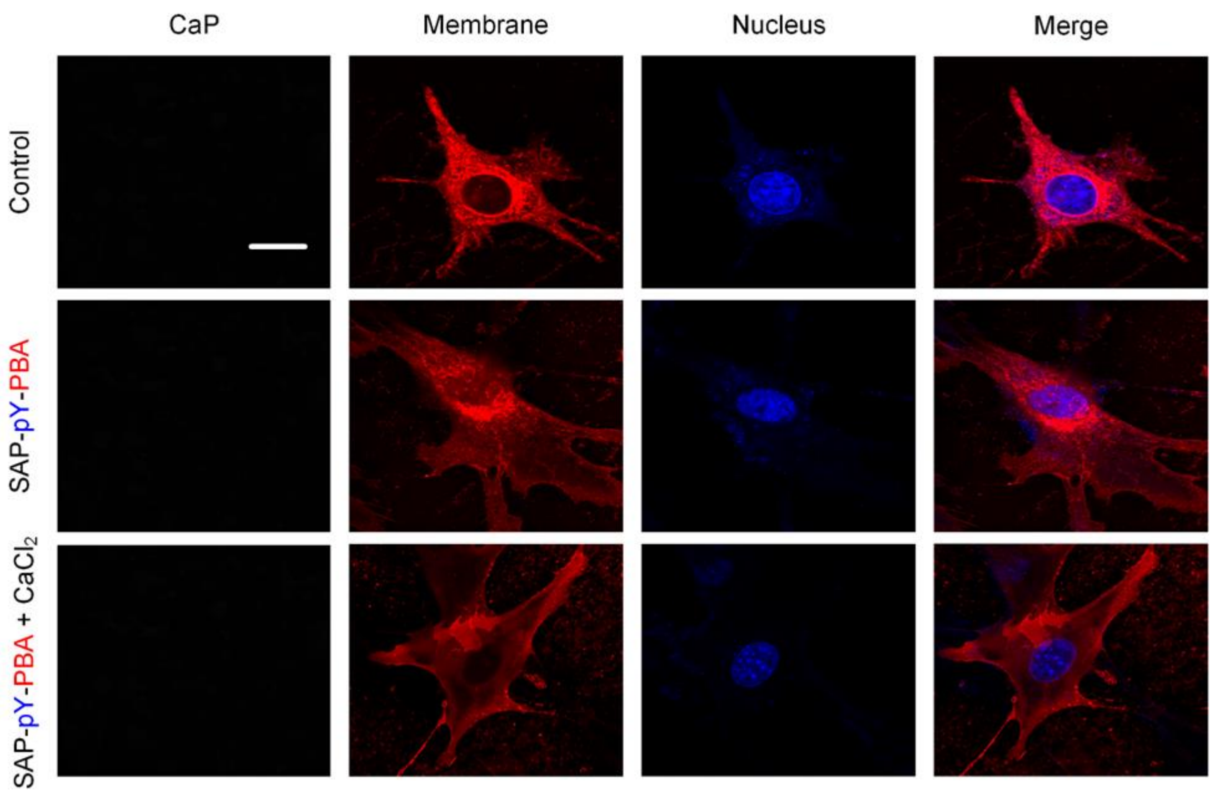

Figure S11. Confocal images of MC3T3-1E cells stained with Calcein (CaP, green), Dil (cell membrane, red) and Hoechst (nuclei, blue) in control, SAP-pY-PBA and SAP-pY-PBA+CaCl$_2$ groups, respectively. Scale bar: 20 μm.

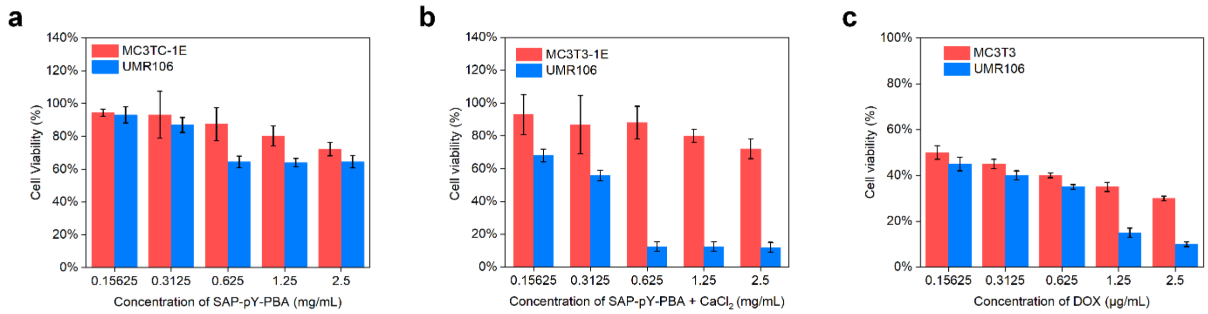

Figure S12. The effect of different concentrations of (a) SAP-pY-PBA, (b) SAP-pY-PBA+CaCl$_2$, and (c) DOX on the viability of MC3T3-1E and UMR106 cells at 48h. Data are expressed as the mean ± SD, n = 5.

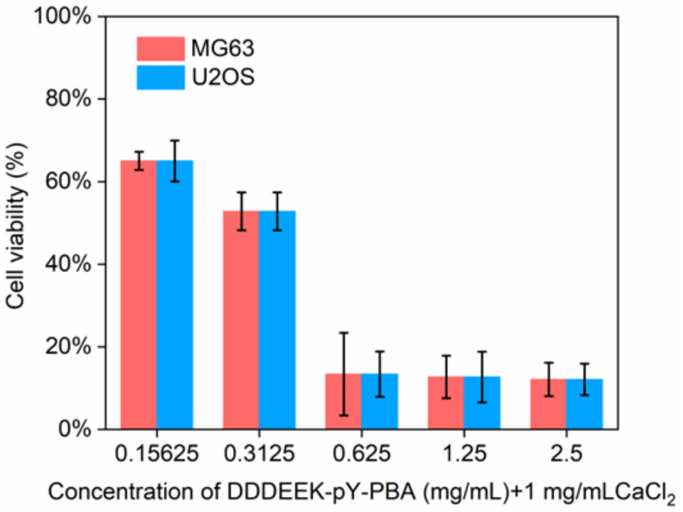

Figure S13. The effect of different concentrations of SAP-pY-PBA+$CaCl_2$ on the viability of human osteosarcoma cell lines MG63 and U2OS at 48h. Data are expressed as the mean ± SD, n = 5.

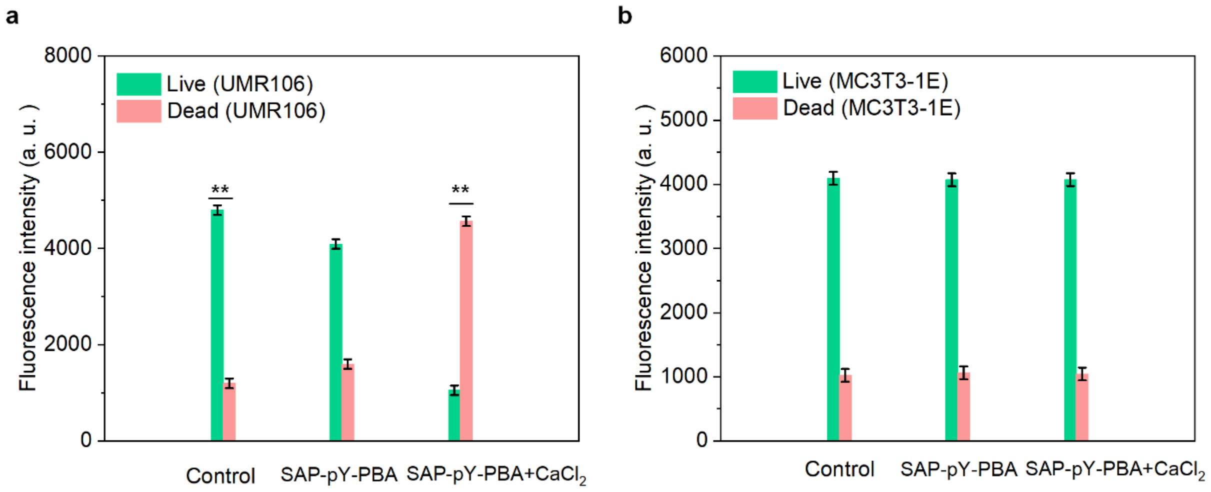

Figure S14. Fluorescence intensity of live/dead staining of UMR106 and MC3T3-1E cells in control, SAP-pY-PBA and SAP-pY-PBA+$CaCl_2$ groups, respectively. Data are expressed as the mean ± SD, n = 5. **p<0.01.

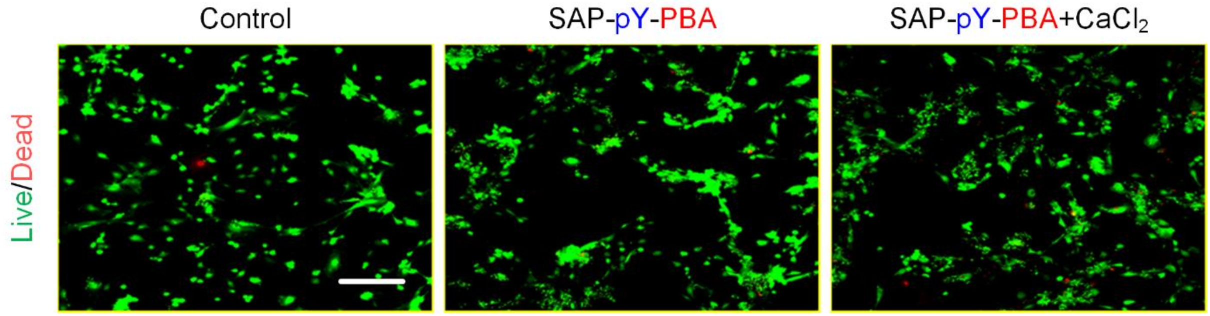

Figure S15. Live/Dead staining of MC3T3-1E cells in control, SAP-pY-PBA and SAP-pY-PBA+$CaCl_2$ groups, respectively. Scale bar: 500 μm.

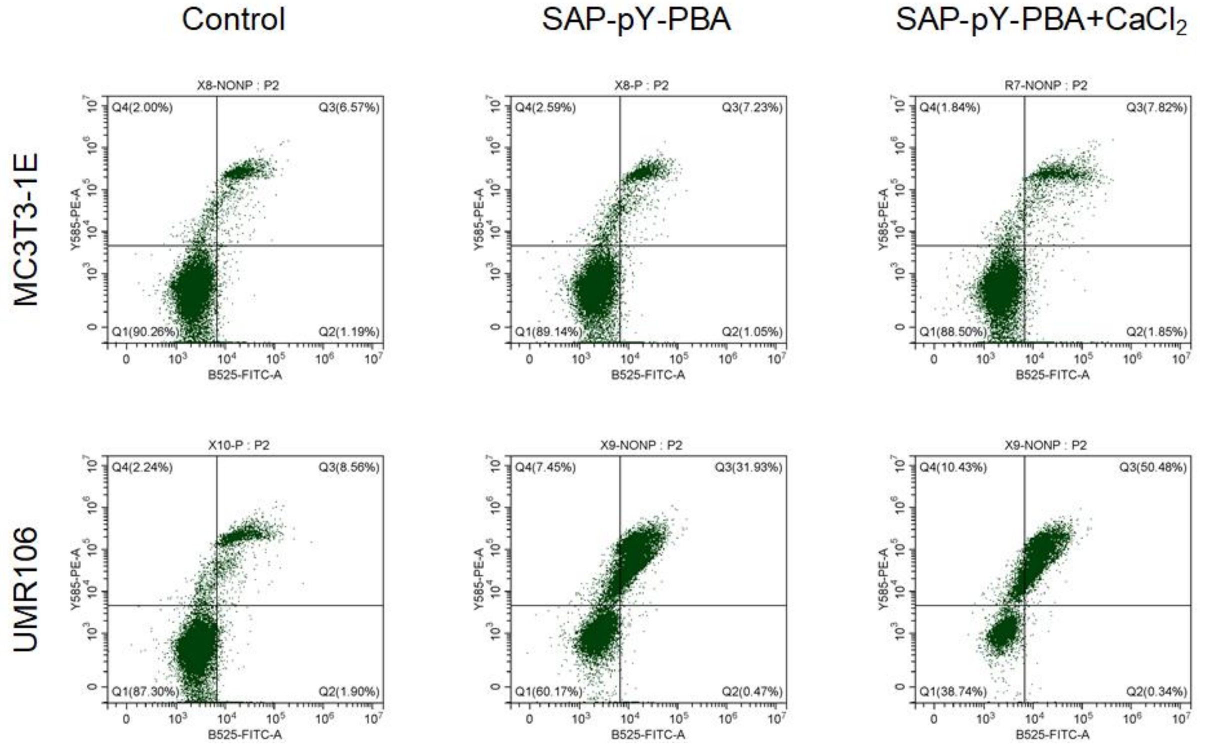

Figure S16. Flow cytometry of MC3T3-1E and UMR106 in control, SAP-pY-PBA and SAP-pY-PBA+$CaCl_2$ group, respectively.

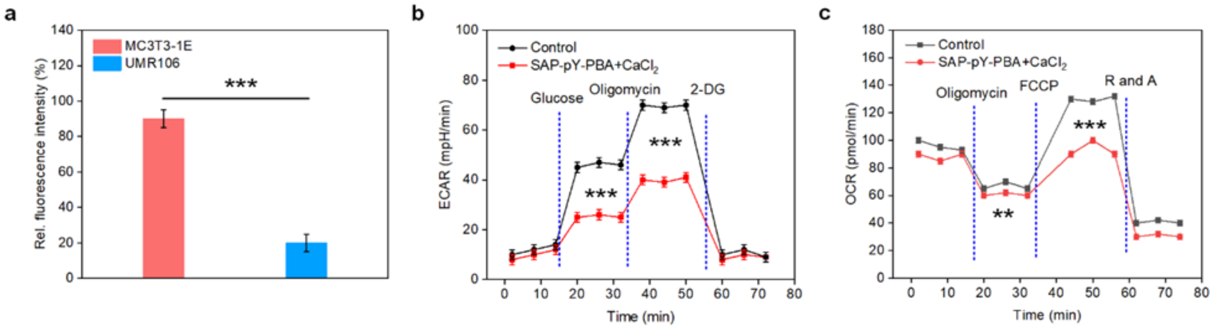

Figure S17. (a) The fluidity of MC3T3-1E and UMR106 cell membranes after SAP-pY-PBA+$CaCl_2$ treatment for 48 h. (b) Glycolytic stress test of control and SAP-pY-PBA+$CaCl_2$ treated UMR106 cells for 48 h. (c) Oxygen consumption rates (OCRs) of isolated mitochondria in control and SAP-pY-PBA+$CaCl_2$ treated UMR106 cells for 48 h. Data are expressed as the mean ± SD, n = 5. $^{**}p < 0.01$, $^{***}p < 0.001$.

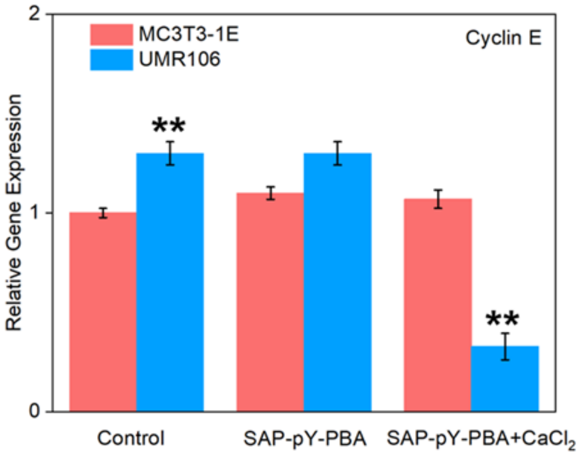

Figure S18. Relative gene expression of Cyclin E in MC3T3-1E and UMR106 cells in control, SAP-pY-PBA and SAP-pY-PBA+$CaCl_2$ groups, respectively. Data are expressed as the mean ± SD, n = 5. ** $p < 0.01$.

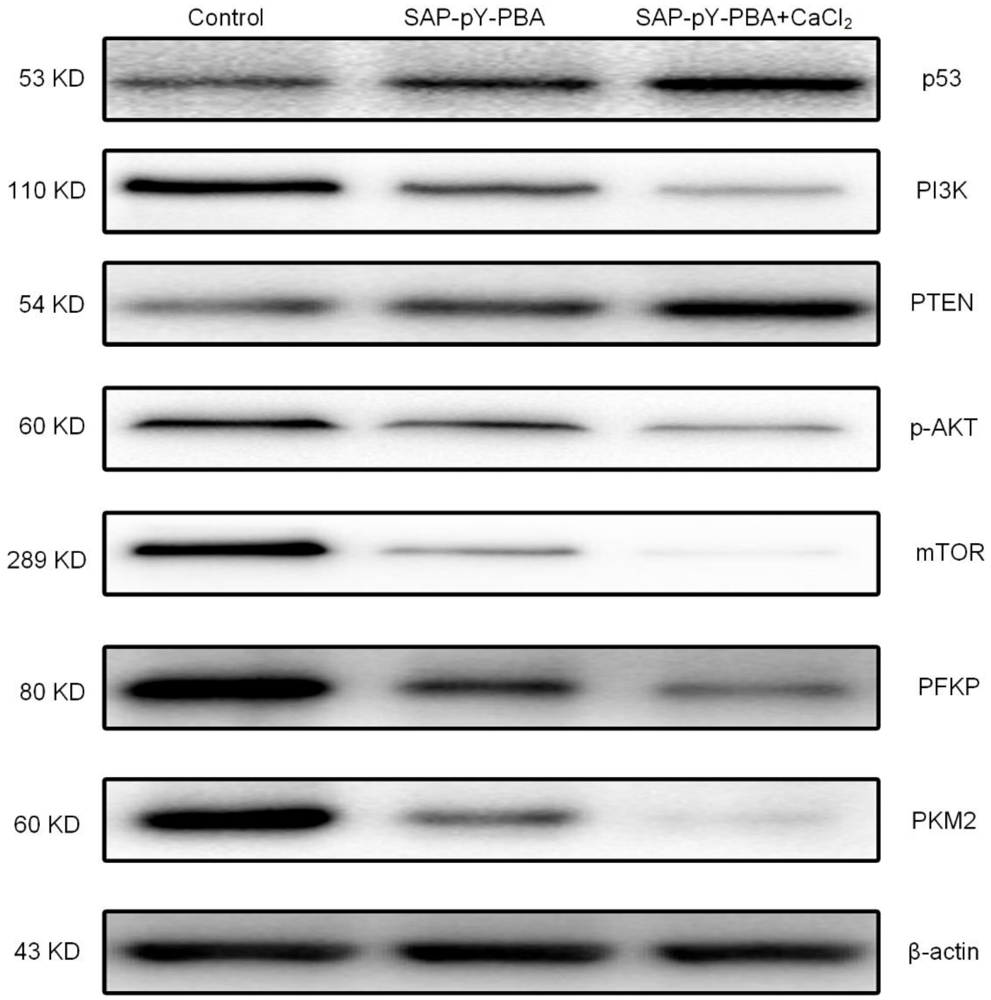

Figure S19. Calcification blocks the cell cycle and inhibits the PI3K/AKT pathway in UMR106 cells.

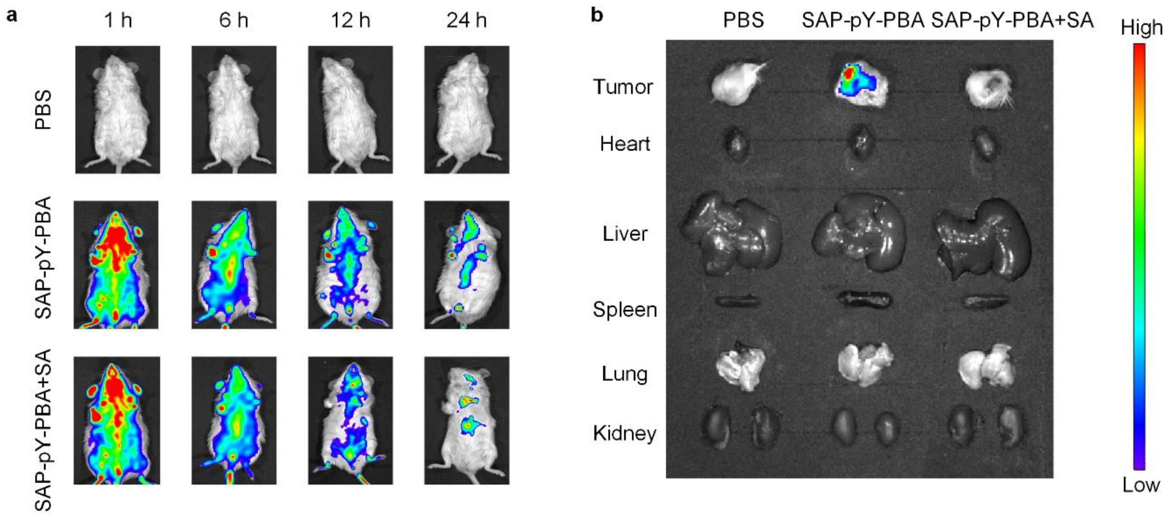

Figure S20. Tumor-target ability detection by *in vivo* imaging (mice) and *ex vivo* imaging (organs); organs included the tumor, heart, liver, spleen, lung, and kidney.

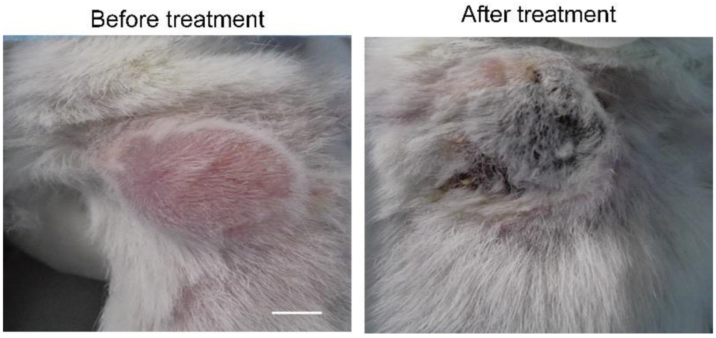

Figure S21. Representative optical images of tumor tissues before and after SAP-pY-PBA+$CaCl_2$ treatment. The tumor tissue calcified and ruptured after treatment. Scale bar = 50 mm.

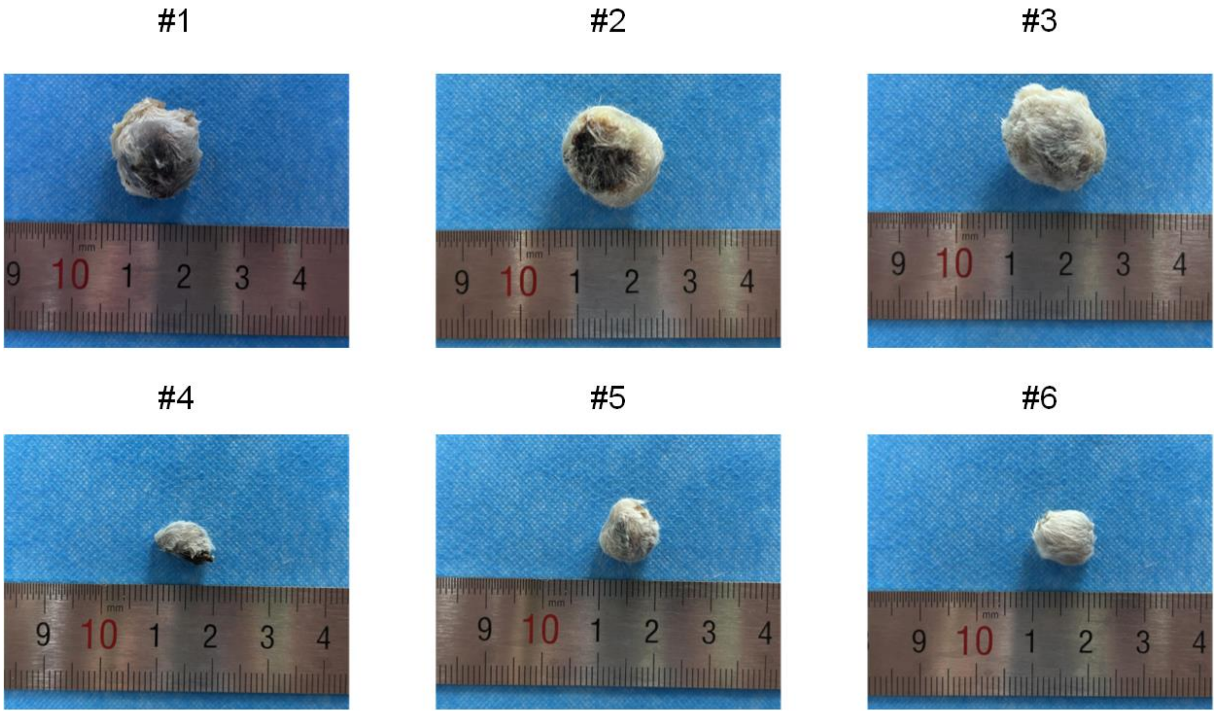

Figure S22. Representative optical images of isolated tumors from body after SAP-pY-PBA+$CaCl_2$ treatment.

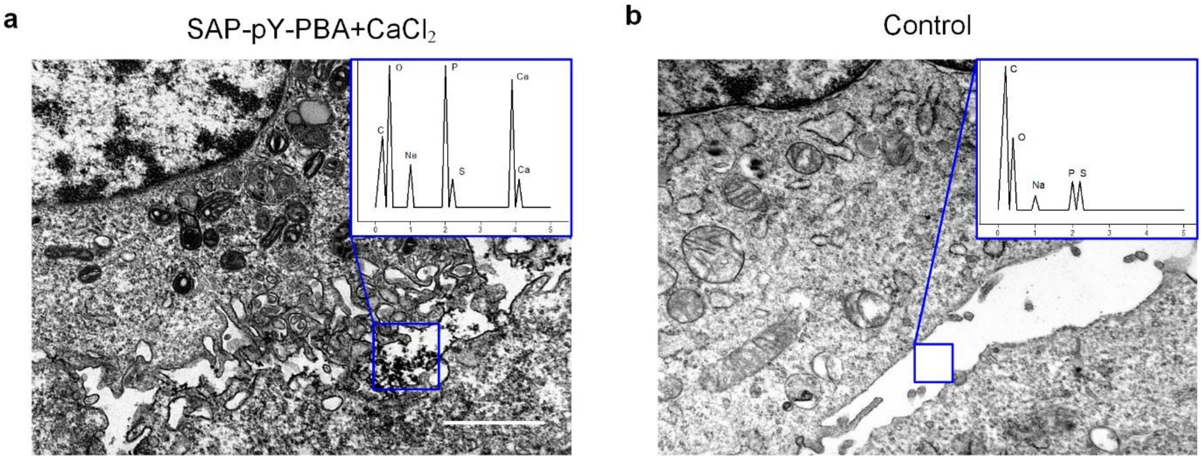

Figure S23. Transmission electron microscopy and EDX (inset) of tumor intercellular calcifications in (a) SAP-pY-PBA+$CaCl_2$ and (b) control group. Scale bar: 2 μm.

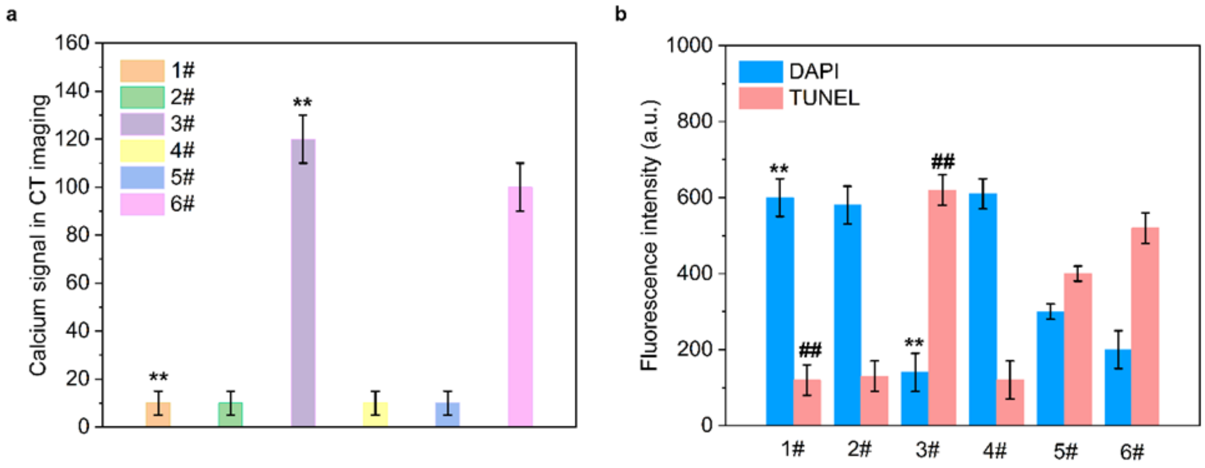

Figure S24. (a) Quantification of calcification signals in tumors. (b) Quantification of fluorescence intensity of a tumor slice stained by DAPI and TUNEL. Data are expressed as the mean ± SD, n = 5. **, ## $p < 0.01$.

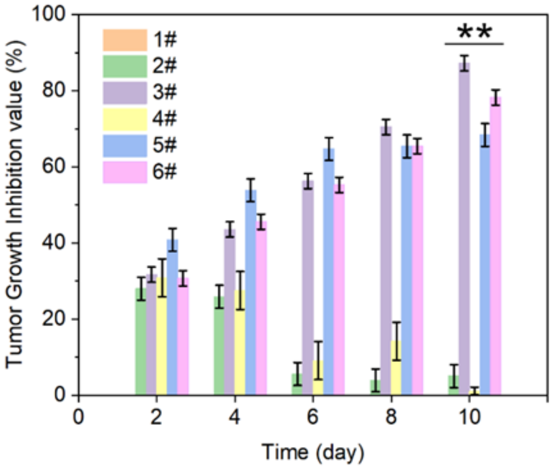

Figure S25. The tumor growth inhibition (TGI) values of UMR106 tumor-bearing mice in the control (1#), SAP-pY-PBA (2#), SAP-pY-PBA+$CaCl_2$ (3#), $CaCl_2$ (4#), DOX (5#) and SAP-pY-PBA via tail vein injection (6#) groups, respectively. Data are expressed as the mean ± SD, n = 5. ** $p < 0.01$.

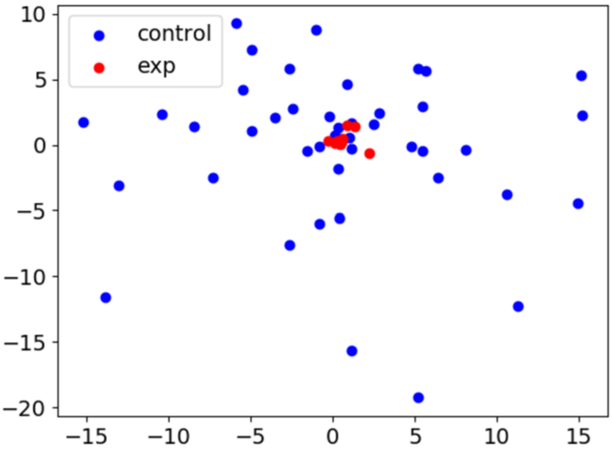

Figure S26. The plot of the visualization of the feature vectors of 100 randomly chosen sub-images using the MDS technique, where 50 data are from the SAP-pY-PBA+$CaCl_2$ class and 50 data are from the "non- SAP-pY-PBA+$CaCl_2$" class. It is noted that since the exp data are too close to each other, it is not apparent the figure actually contains 50 data from this class.

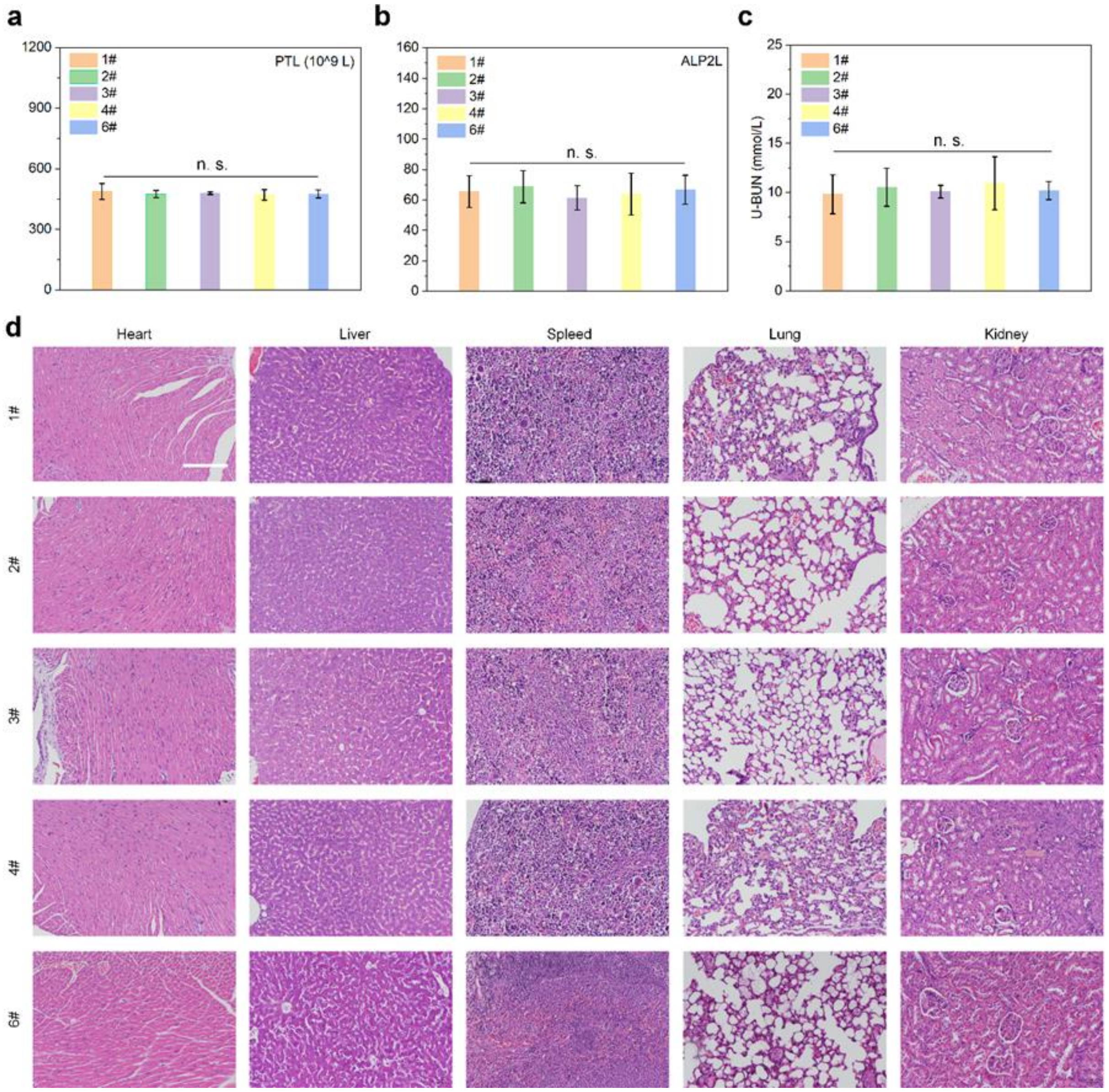

Figure S27. SAP-pY-PBA causes no hematological, blood biochemical, or pathological changes in nontumor tissues in vivo. (a) Hematological analysis of the mice after the different administration treatments. PLT = platelet. (b) Blood biochemistry data of mice in the blood at day 15 post-treatment. (c) BUN = Blood urea nitrogen. (d) Micrographs of H&E staining organ slices from a different group at day 15 post-treatment. Examined organs included the heart, liver, spleen, lung and kidney. These results showed no pathological changes compared with the control (1#), SAP-pY-PBA (2#) and $CaCl_2$ (4#) alone treatment, implying no damage of SAP-pY-PBA+CaCl2 (3#) and SAP-pY-PBA via tail vein injection (6#) to other tissues or organs in the *in vivo* assessments.

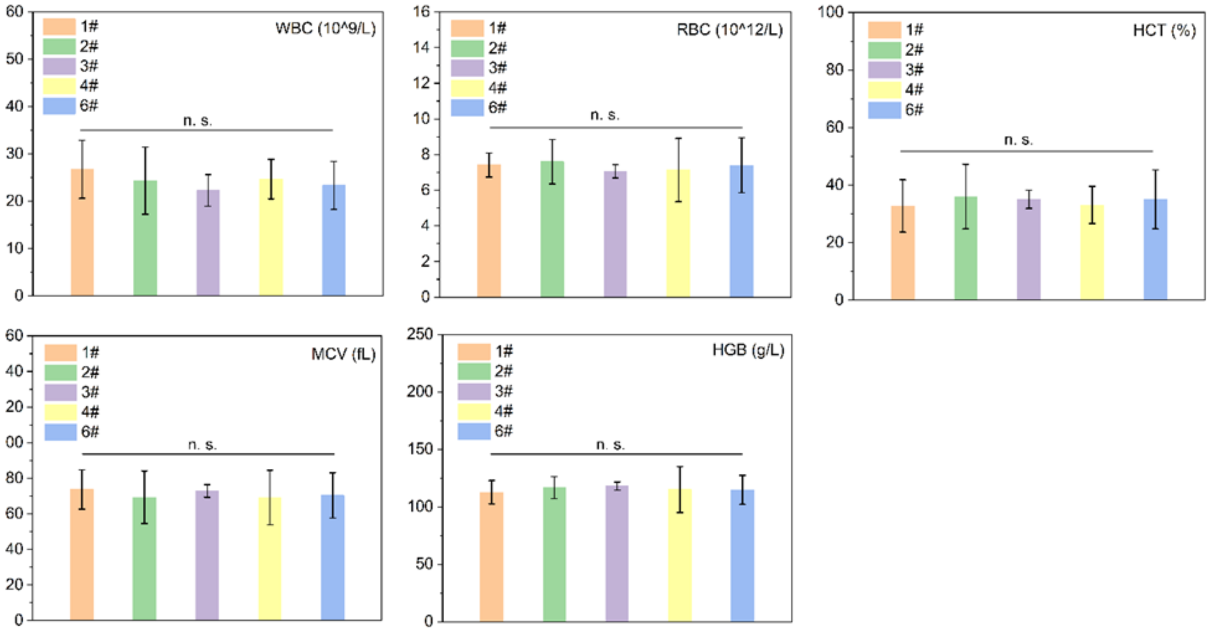

Figure S28. Hematological analysis of the mice after the different administration treatments. WBC = white blood cell, RBC = red blood cell, HCT = hematocrit, MCV = mean corpuscular volume, HGB = hemoglobin. Data are expressed as the mean ± SD, n = 5.

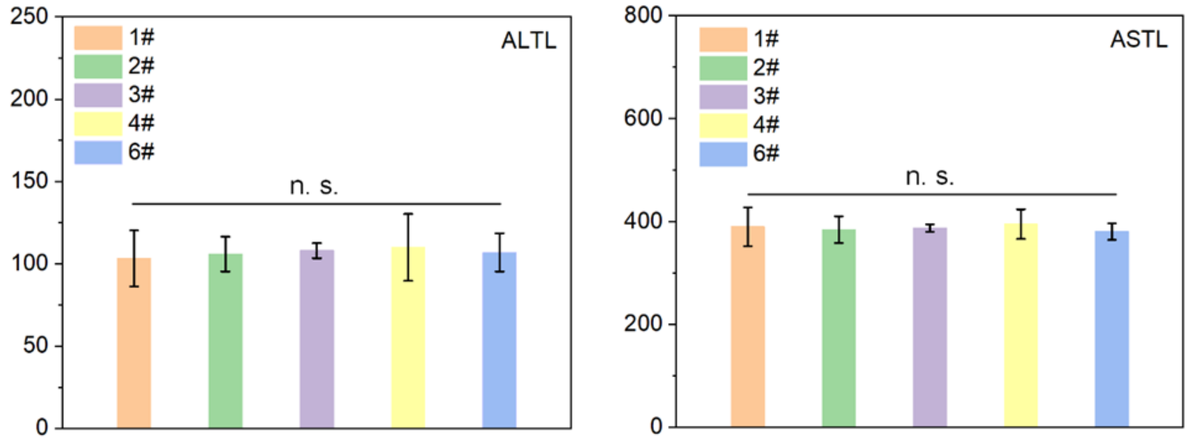

Figure S29. Blood biochemistry data of mice in the blood at day 15 post-treatment, ALP = alkaline phosphatase, AST = aspartate aminotransferase. Data are expressed as the mean ± SD, n = 5.

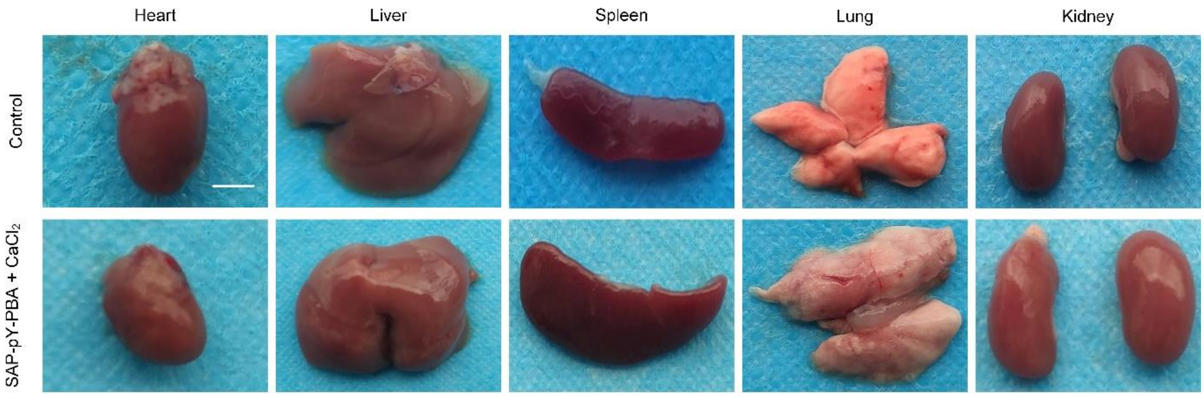

Figure S30. Optical images of heart, liver, spleen, lung and kidney before and after the SAP-pY-PBA+$CaCl_2$ treatment. Scale bar = 0.25 cm.

Table S1. Information of the primers used for real-time PCR (Applied Biosystem).

Gene name	Forward primer	Reverse primer
GAPDH (housekeeping gene)	GTCTCCTCTGACTTCAACAGCG	ACCACCCTGTTGCTGTAGCCAA
ki67	GAAAGAGTGGCAACCTGCCTTC	GCACCAAGTTTTACTACATCTGCC
p27	CCTATTTGGAGAGGTATAGAGTTGT	AGGCATGGCTGAAAAGCAAC
Cyclin D1	TCAAGTGTGACCCGGACTG	CACTACTTGGTGACTCCCGC
Cyclin E	CACAGCTTCGGGTCTGAGTT	GGATGAAAGAGCAGGGGTCC

Table S2. Injection formula for animal experiment.

Numbering	1st dose	2nd dose
#1 group	intratumor injection of 50 µL DMEM	intratumor injection of 50 µL DMEM
#2 group	intratumor injection of 50 µL DMEM containing 1 mg/mL SAP-pY-PBA	intratumor injection of 50 µL DMEM
#3 group	intratumor injection of 50 µL DMEM containing 1 mg/mL SAP-pY-PBA	intratumor injection of 50 µL DMEM containing 1 mg/mL $CaCl_2$
#4 group	intratumor injection of 50 µL DMEM	intratumor injection of 50 µL DMEM containing 1 mg/mL $CaCl_2$
#5 group	intraperitoneal injection of DOX (1mg/kg)	
#6 group	tail vein injection of 50 µL DMEM containing 1 mg/mL SAP-pY-PBA	intratumor injection of 50 µL DMEM containing 1 mg/mL $CaCl_2$

References

[1] a) I. Borg, P. J. F. Groenen, *Modern multidimensional scaling: Theory and applications*, Springer, Berlin, Germany, 2005. b) F. Wickelmaier, *An introduction to MDS*, Sound Quality Research Unit, Aalborg University, Denmark, 2003.
